# Variation in the intestinal microbiota at different developmental stages of *Hynobius maoershanensis*

**DOI:** 10.1002/ece3.8712

**Published:** 2022-03-18

**Authors:** Bo Yang, Zhenzhen Cui, Meihong Ning, Yu Chen, Zhengjun Wu, Huayuan Huang

**Affiliations:** ^1^ Key Laboratory of Ecology of Rare and Endangered Species and Environmental Protection (Guangxi Normal University), Ministry of Education Guilin China; ^2^ Guangxi Key Laboratory of Rare and Endangered Animal Ecology Guangxi Normal University Guilin China

**Keywords:** developmental stage, *Hynobius maoershanensis*, intestinal microbiota

## Abstract

Intestinal microbiota play an important role in the life of amphibians and its composition may vary by developmental stage. In this study, 16S rRNA high‐throughput sequencing was used to profile the intestinal microbiota of *Hynobius maoershanensis*, which exclusively inhabit the Maoer Mountain swamp at an altitude of approximately 2,000 m. We characterized the bacterial composition, structure, and function of the microbiota of *H*. *maoershanensis* at different developmental stages. The alpha diversity was not markedly different for the Simpson, Shannon, Ace, and Sobs indices of microbes. The beta diversity revealed that there were age‐related differences in the structure of the intestinal microbes of *H*. *maoershanensis*, specifically, at the phylum level. Bacteroidetes and Proteobacteria were the dominant bacteria present in the adult stage, and the relative abundance of Bacteroidetes was significantly higher compared with that of tadpoles. Firmicutes and Proteobacteria were the dominant phylum during the tadpole stage and their relative abundance was significantly higher compared with the adult period. Functional analysis revealed that the pathways associated with organismal systems and metabolism were significantly enriched in the adults, whereas human diseases, genetic information processing, and cellular processes were more enriched in the hindlimb bud stage. Human diseases and environmental information processing were more enriched in the forelimb bud stage at KEGG pathway level 1. Possibilities for the observed discrepancies include the adaptation to eating habits and the remodeling of the intestines during development. We speculated that *H*. *maoershanensis* adults may be more suitable to a high‐fiber diet, whereas the tadpoles are associated with a carnivorous diet. Our study provides evidence of variations in the intestinal microbiota during development in amphibians, highlighting the influence of historical developments on the intestinal microbiota and an increased understanding of the importance of physiological characteristics in shaping the intestinal microbiota of amphibians. These data will help us formulate more effective protection measures for *H*. *maoershanensis*.

## Graphical abstract

Hynobius maoershanensis (Urodela: Hynobiidae). The larvae of H. Maoershanensis live an aquatic life, whereas the adults live amphibiously. Due to the sharp decline of its population, it is the key protection wildlife in national nature reserve in China.

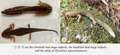

## 1 INTRODUCTION

Vertebrates have a complex symbiotic relationship with their intestinal microbiota, which regulate metabolism, immune function, and energy balance to play a vital role in maintaining health (Hwang et al., 2015; Lapébie et al., 2019). For example, Bacteroidetes, which exist widely in the ecosystem, utilize thousands of enzyme combinations to decompose polysaccharides, convert them into pyruvate and release energy that can be directly used by the host organism (Lapébie et al., 2019; Thomas et al., 2011). Firmicutes, which are widespread in the gastrointestinal tract, can produce byproducts through fermentation, such as short chain fatty acids, that are directly absorbed through the host bowel wall (den Besten et al., 2013). However, higher levels of Bacteroidetes and Firmicutes can increase insulin resistance by modulating the levels of glucagon‐like peptide I, which will lead to a decrease in the efficiency of glucose uptake and utilization (Hwang et al., 2015). Intestinal flora is not always beneficial to the body and can often cause disease. For example, one of the principal risk factors for gastric cancer is Helicobacter pylori infection, which can lead to the accumulation of DNA damage in genes at the ends of chromosome arms, resulting in various genotoxic effects (Koeppel et al., 2015).

Intestinal microbes are affected by many factors. Studies have revealed the relationship between diet and intestinal microbiota; for example, herbivorous and carnivorous animals have different gut microbial characteristics. The intestines of herbivores are dominated by cellulose‐ and lignin‐decomposing bacteria (Rumenococcus, Clostridium, etc.) (Li et al., 2021; Zhang et al., 2019), whereas the intestines of carnivores are mainly concentrated with protein‐ and lipid‐degrading bacteria (Peptostreptococcus) (David et al., 2014; Gillman et al., 2020; Tu et al., 2005). The intestinal microbiota of omnivorous animals, such as wild birds, are primarily associated with Firmicutes (50%) and Proteobacteria (25%) (Kirsten et al., 2018). Feeding habits not only affect the superiority of intestinal bacteria but also the abundance. In a study of intestinal microbes based on nine diets of 62 species of insects, those that fed on rotted wood had the highest abundance of gut microbes, whereas insects that fed on pollen wood exhibited the lowest (Colman et al., 2012). The intestinal microbiota are also related to changes in dietary resources. When aliments were plentiful, Ruminococcaceae (90%) were the main species of the African buffalo; however, under food restriction conditions, Solibacillus (72%) was the predominant species (Couch et al., 2021). Marked differences in the structure of intestinal microbiota caused by dietary transformation are also reflected in amphibians; with gastropods as the primary food source, the intestinal microbiota of fire salamanders were Bacteroidea (47.8%) and Firmicutes (32.1%) (Wang et al., 2021), whereas the intestinal microbiota were primarily Proteobacteria and Firmicutes in tadpoles which are mainly phytophagous and that of insectivorous adults were mainly Firmicutes and Bacteroides (Kohl et al., 2013). These studies suggest that the abundance of intestinal microbiota is closely related to diet.

Age is closely related to the intestinal microbiota of animals. The reorganization of intestinal microbiotas caused by aging is primarily related to changes in the structure and physical/chemical properties of the digestive system (Bonder et al., 2016; Chen, Cui, et al., 2021; Chen, Li, et al., 2021; Vangay et al., 2018; Zhang et al., 2021). For instance, the stripping and shedding of old intestines during the metamorphic development of some mosquitoes can lead to complete or nearly complete elimination of intestinal bacteria, resulting in the absence of bacteria in the intestines of newly emerged adults (Moll et al., 2001). Amphibians complete the transformation of their digestive tracts from a long and simple non‐acidic stomach to a short and complex acidic stomach through a metamorphosis process (Zhang et al., 2020). The close relationship between the structure of the digestive tract and dietary changes can lead to changes in the intestinal microbiomes (Schreiber et al., 2005). Studies have shown that the intestinal microbiota of frogs change from fish‐like (Proteobacteria, Firmicutes) during the tadpole stage to an amniotic membrane (Firmicutes, Bacteroides) phenotype during the frog stage (Kohl et al., 2013; Zhang et al., 2018). In addition, changes in age result in altered metabolic levels, which affect the structure of the intestinal microbiomes. For example, a Japanese study showed that people over 100 years old contain higher levels of secondary bile acid (produced by intestinal bacteria metabolism) compared with 40‐year‐olds and those under the age of 100. This significantly reduces the risk of aging diseases compared with those of the elderly under 100 years old (Rimal & Patterson, 2021).

A large number of studies involving microbiota have focused on mammals and fish (Frese et al., 2015; Li et al., 2021; Sullam et al., 2012), whereas studies on amphibians have been primarily focused on tailless amphibians (Chai et al., 2018; Kohl et al., 2013; Vences et al., 2016; Weng et al., 2016). Few studies have been conducted on the intestinal microbiotas of tailed amphibians in populations living under artificial feeding conditions (Zhang et al., 2018). There have been a few studies on the intestinal microbiota of wild populations, especially tailed amphibians that are rare species. Currently, amphibians are suffering from a rapid decline and are most susceptible to diseases in their ecosystem (Greener et al., 2020). Because amphibians play important roles in the ecosystem, studying their symbiotic microbiota may lead to strategies for their protection (Alford, 2011; Vences et al., 2016). Therefore, it is necessary to expand intestinal microbiota studies with more species.

*Hynobius maoershanensis* primarily lives in the surrounding area of the alpine swamp and the vegetation nearby consists primarily of hemlock forest and mountaintop dwarf forest (Chen, Cui, et al., 2021; Chen, Li, et al., 2021; Jiang & Jiang, 2006). The larvae of *H*. *maoershanensis* live an aquatic life, whereas the adults live amphibiously. Their diets and digestive systems undergo significant changes during development (Chen, Cui, et al., 2021; Chen, Li, et al., 2021; Ning et al., 2021). Based on the significant changes during development, we hypothesized that there are distinct differences in the structure and function of the intestinal microbiota during the three developmental stages (forelimb bud stage tadpoles, hindlimb bud stage tadpoles and adults) of the tailed amphibian, *H*. *maoershanensis*. We identified the influencing factors of the intestinal microbes in the ontogeny of *H*. *maoershanensis*, which result in changes in the intestinal microbiota during the development of amphibians, especially tailed amphibians. Our findings provide a reference for monitoring the health and physiological conditions of wild amphibians.

## 2 MATERIALS AND METHODS

### 2.1 Sample collection and preservation

*Hynobius maoershanensis* belongs to the *Hynobius hynobius* genus and only lives in an area around the high mountain marshes of Xingan County, Guangxi Zhuang Autonomous Region (25°52′N, 110°24′E) at an altitude of 1,950–2,000 m.

Samples were obtained from the cloaca of 31 adults, 12 hindlimb bud tadpoles, and 30 forelimb bud tadpoles of *H*. *maoershanensis* in December 2019 (Table 1). Anal swabs were collected by nondestructive sampling. The cloaca was wiped with an alcohol swab before inserting a sterile cotton swab and rotating for three to five circles and then placed into a sterile preservation tube (Colston et al., 2015). All samples were frozen immediately after collection, transported to the laboratory, and stored at −80℃ (Song et al., 2018). All *H*. *maoershanensis* were returned to their original location after sampling.

**TABLE 1:** Host information of 73 anal swab samples of *H*. *maoershanensis*

	Adult	Hindlimb bud stage	Forelimb bud stage
Total length (mm)	165.04 ± 12.42	63.96 ± 7.88	27.42 ± 36.61
Weight (g)	21.09 ± 5.00	1.82 ± 0.67	0.23 ± 0.17

### 2.2 DNA extraction, amplification, and sequencing

Total bacterial genome DNA from all samples was extracted using the E.Z.N.A.^®^ Soil DNA kit (Omega Bio‐tek). The V3–V4 hypervariable region of the 16S rRNA gene was amplified by the polymerase chain reaction (GeneAmp 9700; ABI) using the general bacterial primers (338F, 5′‐ACTCCTACGGGAGGCAGCAG‐3′; 806R, 5′‐GGACTACHVGGGTWTCTAAT‐3′) (Mori et al., 2014). The initial PCR was performed using Transgen ap221–02 TransStart^®^ FastPfu Fly DNA Polymerase. A 20 μl reaction mixture contained 10 ng template DNA, 4 μl 5× FastPfu buffer, 2 μl 2.5 mM dNTPs, 0.8 μl of each primer (5 μM), and 0.2 μl bovine serum albumin. The PCR products were extracted from a 2% agarose gel and purified using an AxyPrep DNA Gel Extraction Kit (Axygen Biosciences). The purified PCR fragments were collected and adjusted to an equal molar concentration (Majorbio BioPharm Technology Co., Ltd, Shanghai provide sequencing services) and the paired ends were sequenced (2 × 300) using an Illumina Miseq platform (Illumina).

### 2.3 Data analysis

On the basis of the overlap between PE reads, the paired reads were merged into a single sequence. The quality of the reads and the effect of the merge were filtered by in a quality control step. According to the barcodes and primer sequences at the beginning and end of the sequence, the effective sequences were obtained and the sequence direction was corrected. The data were flattened according to the minimum number of sample sequences. The base was filtered if the tail mass value was less than 20 and set to a window of 50 bp. The back‐end base was cut from the window if the average quality value in the window was lower than 20. The reads below 50 bp were filtered after quality control and the reads containing an “N” base were removed. The minimum overlap length was 10 bp, the maximum mismatch ratio of the overlap region was 0.2, the allowable mismatch number of the barcode was 0 and the maximum mismatch number of the primers was 2 (Trimmomatic software, Illumina) (Raman et al., 2019).

The original fastq file uses flash for pair‐end double‐ended sequence splicing. Uparse (version number: 7.0.1090; http://www.drive5.com/uparse/) operational taxonomic unit (OTU) clustering and Qiime (version number: 1.9.1; http://qiime.org/install/index.html) were used. The water abundance table for each taxonomic species was generated and the beta diversity distance was calculated. An RDP classifier (version 2.11; https://sourceforge.net/projects/rdp‐classifier/) was used for sequence classification. Using the Greengenes database (version 135; http://greengenes.secondgenome.com/), each 16S rRNA gene sequence was classified using rRNA database alignment.

The alpha diversity indices (Shannon index, Simpson index, ACE index, and Chao index) were calculated using the mothur program (version v.1.30.1; http://www.mothur.org/wiki/Schloss_SOP#Alpha_diversity). The diversity distance matrix of beta was calculated by Qiime (http://qiime.org/install/index.html). Principal coordinate analysis (PCoA) was used to calculate and visualize weighted and unweighted UniFrac distance matrices. Nonparametric Anosim analysis was used to test whether the difference between groups was significantly greater compared with that within groups. The Wilcoxon rank‐sum test and FDR‐adjusted *p* values were used to test the difference of genes, and KEGG pathways (http://picrust.github.io/picrust/) were compared between groups to predict their function. All data were analyzed using the Majorbio I‐Sanger Cloud Platform (http://www.i‐sanger.com).

## 3 RESULTS

### 3.1 Sequence quality evaluation

**FIGURE 1:**
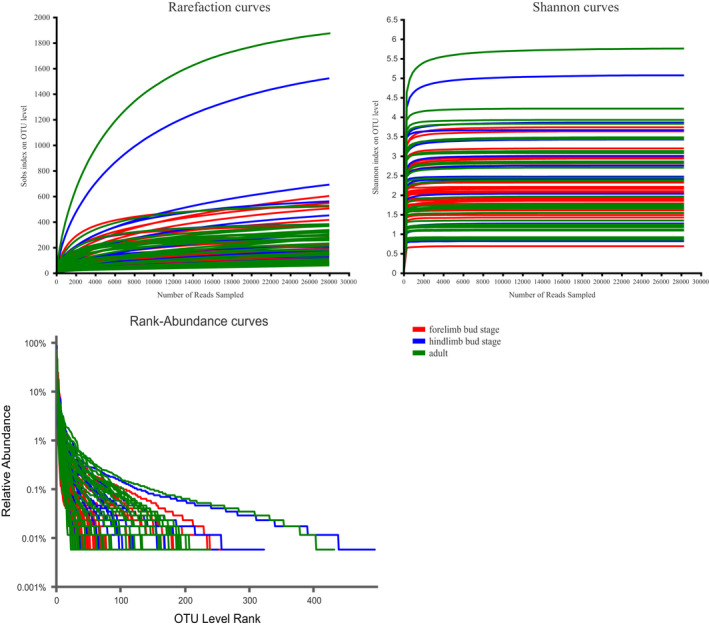
Rarefaction curves and rank‐abundance distribution curves

A total of 3,329,196 sequences of the hypervariable V3–V4 region of the 16S rRNA gene were obtained from 73 fecal samples, of which 1,073,747 were valid, and the average length of the sequences were 417 bp. The sample sequences were leveled in accordance with the minimum number of sample sequences (CD14:28144). A total of 1,035 OTUs were clustered using a sequence similarity of 97%. The rank abundance, rarefaction, and alpha diversity index was constructed based on these OTUs and showed that the depth of the sequencing results was sufficient (Figure 1; Table 2).

**TABLE 2:** Alpha diversity index of *H*. *maoershanensis* gut microbiota (a: forelimb bud stage and hindlimb bud stage b: forelimb bud stage and adults c: hindlimb bud stage and adults)

	Hindlimb bud stage	Forelimb bud stage	*p*‐value	*Q*‐value (FDR)
(a)				
Sobs	19.167 ± 9.98	14.467 ± 5.847	.108	0.396
Shannon	0.932 ± 0.529	0.799 ± 0.262	.237	0.396
Simpson	0.551 ± 0.255	0.569 ± 0.151	.351	0.396
Ace	21.437 ± 10.5	19.768 ± 15.004	.396	0.396

	Adult	Forelimb bud stage	*p*‐value	*Q*‐value (FDR)
(b)				
Sobs	15.419 ± 7.898	14.467 ± 5.847	.558	0.558
Shannon	0.959 ± 0.343	0.799 ± 0.262	.337	0.531
Simpson	0.475 ± 0.129	0.569 ± 0.151	.030	0.120
Ace	20.744 ± 10.651	19.768 ± 15.004	.399	0.531

	Adult	Hindlimb bud stage	*p* value	*Q*‐value (FDR)
(c)				
Sobs	15.419 ± 7.898	19.167 ± 9.98	.188	0.751
Shannon	0.959 ± 0.343	0.932 ± 0.529	.989	1.000
Simpson	0.475 ± 0.129	0.551 ± 0.255	1.000	1.000
Ace	20.744 ± 10.651	21.437 ± 10.5	.807	1.000

### 3.2 Analysis of alpha and beta diversity

**FIGURE 2:**
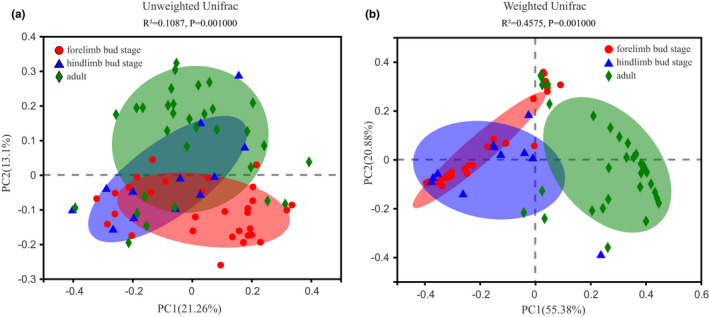
Beta diversity difference in gut microbiota at different developmental stages. (a) unweighted‐unfair; (b) weighted‐infair

The alpha diversity analysis, based on 1035 OTUs (Table 2), revealed that there were no significant differences in the Shannon, Simpson, ACE, and Sobs indices between the three stages (FDR > 0.05) (Table 2), which indicates a similar diversity. PCoA based on unweighted (*R* ^2^ = 0.109, FDR < 0.001) and weighted (*R* ^2^ = 0.458, FDR < 0.001) UniFrac distances by the Adonis test showed that the intestinal microbiota were highly aggregated by different developmental stages. This suggests that there were differences in the structure of the intestinal microbiota in *H*. *maoershanensis* (Figure 2).

### 3.3 Difference in the composition and structure of intestinal microbiota

**FIGURE 3:**
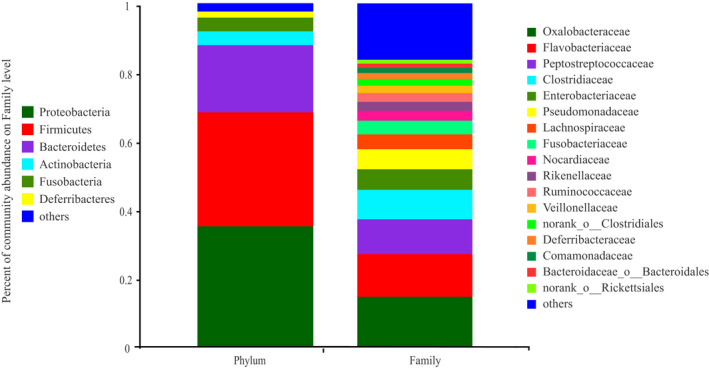
Gut microbiota composition at the phylum and family level

**FIGURE 4:**
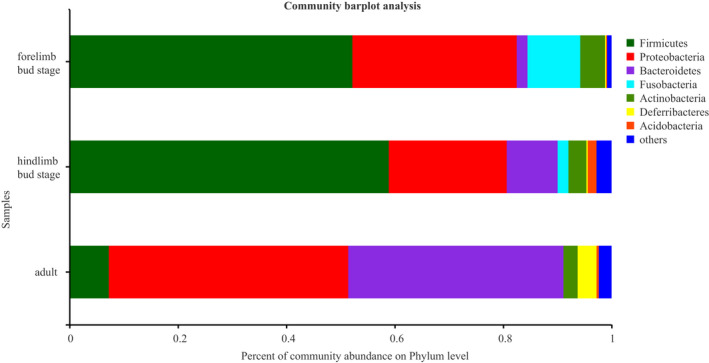
Gut microbiota composition at the phylum level

**FIGURE 5:**
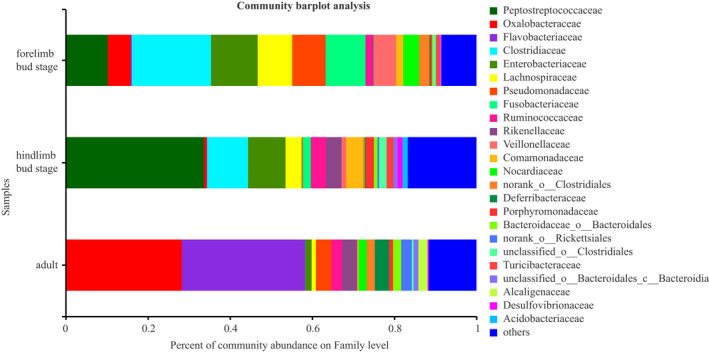
Gut microbiota composition at the family level

**FIGURE 6:**
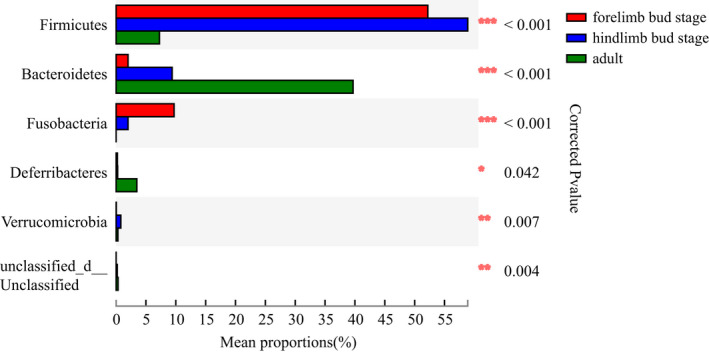
The gut microbiota composition and difference at the phylum level at different developmental stages

**FIGURE 7:**
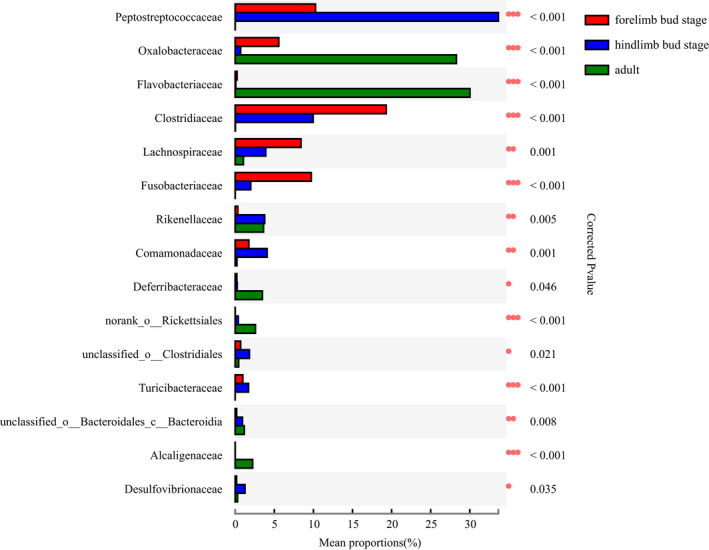
The gut microbiota composition and difference at the family level at different developmental stages

The OTUs (*n* = 1,035) obtained from the samples consisted of 29 phyla and 214 microbial families. The most dominant phyla were Proteobacteria (35.04%), Firmicutes (33.19%), and Bacteroidetes (19.57%). The most dominant families were Oxalobacteraceae (14.46%), Flavobacteriaceae (12.43%), and Peptostreptococcaceae (10.15%) (Figure 3). Considering the different stages of development, the proportion of intestinal microbiotas differed remarkably in the three stages. At the phylum level, the top three phylum with the highest enrichment of forelimb bud stage were Firmocutes, Proteobacteria, and Fusobacteria, whereas those in the hindlimb bud stage and adults were Firmicutes, Proteobacteria, and Bacteroidetes (Figure 4). At the family level, the top three families with the highest enrichment in the forelimb and hindlimb bud stage were Peptostreptococcaceae, Clostridiaceae, and Enterobacteriaceae, whereas the adults consisted of Oxalobacteraceae, Flavobacteriaceae, and Pseudomonadaceae (Figure 5). Based on a Wilcoxon rank‐sum test, the relative abundance of Firmicutes and Fusobacteria in the tadpole stages were markedly different compared with that in the adult stage (FDR < 0.001), whereas Bacteroidetes were more enriched in adults (FDR < 0.001) at the phylum level (Figure 6). At the family level, Peptostreptococcaceae was more enriched in the tadpole stage compared with the adults, especially in the hindlimb bud stage (FDR < 0.001); Clostridiaceae were enriched in the tadpole stage compared with the adults, especially in the forelimb bud stage. In contrast, the relative abundance of Oxalobacteraceae and Flavobacteriaceae in the adults was significantly higher than that in tadpoles (FDR < 0.001) (Figure 7). The relative proportions of the other bacterial phyla and families are listed in Appendices S1 and S2.

**FIGURE 8:**
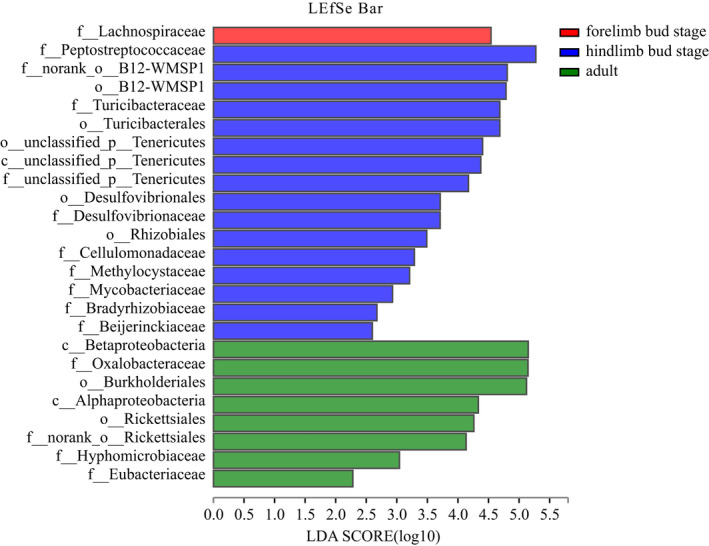
The LEfSe of gut microbiota abundance

Lefse analysis was used to detect differences in the relative abundance of microbiota at different levels to further identify shifts in composition at different stages. The results showed that there were 25 intestinal bacterial taxa with higher abundance in this group of which one taxa was from the forelimb bud stage, 16 were from the hindlimb bud stage, and eight were from the adults. The Peptostreptococcaceae family, the Betaproteobacteria class, the Oxalobacteraceae family, and the Burkholderiates order were the major taxa contributing to these differences (Figure 8).

### 3.4 Differences in the functional distribution of intestinal flora

The Mann–Whitney *U* test was used to identify differences in KEGG pathways to predict the function of the intestinal microbiota at different developmental stages. Pathways associated with organismal systems and metabolism were significantly enriched in adults, whereas genetic information processing and cellular processes were more enriched in the hindlimb bud stage. Human diseases and environmental information processing were more enriched in the forelimb bud stage at KEGG pathway level 1 (Table 3). For KEGG pathway level 2, pathways associated with amino acid metabolism, cancer, carbohydrate metabolism, and cardiovascular disease were significantly enriched in adults, whereas pathways associated with biosynthesis of other secondary metabolites were enriched in the hindlimb bud stage (Appendix S3).

**TABLE 3:** Differences in KEGG Pathways Level 1

Pathway in level 1	Relative abundance (%)			Mann–Whitney *U* test	
	Forelimb bud stage	Hindlimb bud stage	Adult	*z*	*p* (FDR)
Metabolism	38.43 ± 0.36	37.06 ± 0.38	40.12 ± 0.37	−4.631	.000
Human Diseases	25.58 ± 0.03	24.99 ± 0.03	24.84 ± 0.03	−4.501	.000
Genetic Information Processing	15.57 ± 0.40	18.04 ± 0.49	15.64 ± 0.43	−4.386	.000
Environmental Information Processing	16.01 ± 1.24	15.27 ± 1.14	15.1 ± 1.21	−3.073	.002
Cellular Processes	3.64 ± 0.23	3.92 ± 0.25	3.48 ± 0.22	−4.111	.000
Organismal Systems	0.77 ± 0.05	0.72 ± 0.04	0.82 ± 0.05	−3.982	.000
Unclassified	0.00 ± 1.01	0.00 ± 1.03	0.00 ± 0.94	−4.645	.000

## 4 DISCUSSION

Based on our study, the beta diversity analysis of the intestinal microbiota of *H*. *maoershanensis* showed differences in age, whereas the alpha diversity revealed a higher degree of similarity. The results were similar to that of the northern leopard frog (*Lithobates pipiens*) (Kohl et al., 2013). Adults of *H*. *maoershanensis* exhibited a greater abundance of Oxalobacteraceae and Flavorbacteriaceae. In terms of function, the organic systems and metabolism pathways were significantly enriched. The hindlimb bud stage exhibited a higher abundance of Peptostreptococcaceae, and the functional pathways of genetic information processing and cellular processes were enriched, whereas the forelimb bud stage contained a higher abundance of Clostridiaceae and Enterobacteriaceae. In terms of predicting function, the human diseases, environmental information processing approach was more enriched. The differences of structure and function of intestinal microbiotas of *H*. *maoershanensis* based on the different developmental stages were consistent with our predictions and similar results have been reported for other amphibians, such as the northern leopard frog (*Lithobates pipiens*) (Kohl et al., 2013), *Bufo gargarizans* (Chai et al., 2018), which may be related to age and age‐based dietary changes. Generally, in mammals, amphibians, or even some insects, gut microbes will undergo morphological, physical, and chemical changes concomitantly with the development of the digestive system (Colman et al., 2012; Kohl et al., 2013; Ley et al., 2008; Moll et al., 2001). During the development of amphibians, there will be changes in the living environment (from completely aquatic to terrestrial life) and adults exhibit a higher diversity and richness of intestinal microbiota compared with tadpoles (Chai et al., 2018; Kohl et al., 2013). However, the alpha diversity of adults and tadpoles did not show significant differences, which may be related to the sampling time of adults. We sampled *H*. *maoershanensis* during underwater breeding activities. At this time, the habitat of the adult is consistent with that of the tadpole. As with other species, there was no significant difference in the alpha diversity of intestinal microbiotas at different stages of ontogeny and the reasons for this may be diverse (Chen, Cui, et al., 2021; Chen, Li, et al., 2021; Kohl et al., 2013).

At present, there have been no definitive studies on the feeding habits of *H*. *maoershanensis*. Studies have shown a decrease in the Firmicutes:Bacteroidetes ratio during the adaptation to a high‐fiber diet, whereas an increase in the ratio tends result from high‐protein food consumption (De Filippo et al., 2010). In this study, the abundance of Firmicutes (adult: 7.27% ± 11.67%, hindlimb bud stage: 58.9% ± 28.41% and forelimb bud stage: 52.19% ± 31.03%) and Bacteroides (adult; 39.69% ± 21.4%, hindlimb bud stage: 9.38% ± 16.37%, and forelimb bud stage: 1.99% ± 2.7%) were significantly different at different developmental stages (*p* < .001, FDR < 0.001). The Firmicutes:Bacteroidetes ratio was the highest during the forelimb bud stage (26.226), followed by the hindlimb bud stage (6.279), and the adult stage (0.183). In addition, there was a higher abundance of Oxalobacteriaceae in the adult intestines (28.28) (*p* < .001, FDR < 0.001), a type of bacteria that mainly digests oxalic acid which is enriched in plant foods (Green et al., 2007; Yu et al., 2021). In addition, the adult intestine had a significantly higher Bacteroide content (39.69% ± 21.4%) (*p* < .001, FDR < 0.001) compared with the tadpole stages, because the bacteria in this phylum have the ability to degrade and utilize host mucin glycosides, especially when there is a lack of food‐derived substrates in the intestine. These are considered bacteria that are adapted to food‐deficient environments (Flint et al., 2012; Sonnenburg et al., 2005). Therefore, it is speculated that the increase of Bacteroides in the adult intestine of *H*. *maoershanensis* may result from a nutrient‐poor environment. Based on these factors, we speculate that the *H*. *maoershanensis* adults may be more adapted to a high‐fiber diet with a higher proportion of plant foods. The adult *H*. *maoershanensis* may be facing a severe shortage of food. Compared with adults, for the sake of the nutritional requirements for maximum growth of the organism during the tadpole stage, the intestinal microbiotas of the tadpole exhibited higher levels of Peptostreptococcaceae, Clostridiaceae and Fusobacteriaceae (Soverini et al., 2016), which have higher proteolysis and amino acid metabolism activities, indicating that *H*. *maoershanensis* in the tadpole stage may prefer a carnivorous diet. To some extent, this is consistent with the view that tadpoles have a diverse diet (Bennett, 1992; Karasov, 1992). The functional differences of intestinal microbiota at different developmental stages might be attributed to the changes of diet and development (Chen, Cui, et al., 2021; Chen, Li, et al., 2021; Moya & Ferrer, 2016). For example, the relative abundance of Clostridiaceae was more enriched in the forelimb bud stage, the infections with bacteria of the genus *Sarcina* (family Clostridiaceae) in human and animal were associated with gastric dilation and emphysematous gastritis (Owens et al., 2021), consistent with the functional prediction that human disease were more enriched in the forelimb bud stage. The prediction of intestinal microbial function of *H*. *maoershanensis* needs to be further studied.

There are two points worth noting. First, *H*. *maoershanensis* adults contain a significantly higher abundance of Flavobacteriaceae (up to 30%) compared with the tadpole stages. Second, there was no significant difference in the relative abundance of Pseudomonadaceae during the three developmental stages, which accounted for a high proportion in each group (Adult: 3.78% ± 10.7%, hindlimb bud stage: 0.28% ± 0.5%, and forelimb bud stage: 8.13% ± 20.07%). Flavobacterium are pathogens associated with cold water disease worldwide (Kumru et al., 2020). They can cause diseases, such as red‐skin disease and ulcers. Pseudomonadaceae can cause septicemia and rot in aquatic animals (Miklos et al., 1999). These bacteria act on target cells by secreting proteins that interfere with their normal function to cause pathogenicity (Abby et al., 2016). Studies have shown that amphibians exhibit poor resilience after infection from such pathogens (Jani et al., 2021). Whether these two pathogenic bacteria cause widespread infection of *H*. *maoershanensis* and population decline remains to be determined.

In summary, the structure and function of the intestinal microbiota of *H*. *maoershanensis* at different developmental stages are significantly different. Future studies will expand on the information gathered on the intestinal microbiota of *H*. *maoershanensis*, including whether gender and reproduction period affect the structure and diversity of intestinal microbiota. Since our research was significantly restricted, more behavioral observations and other studies will be carried out in later stages, such as the maintenance and improvement of population numbers through the study of activities, environment and social habits in *H*. *maoershanensis* during nonreproductive periods.

## CONFLICT OF INTEREST

The authors declare no conflicts of interest.

## AUTHOR CONTRIBUTIONS

**Bo Yang:** Data curation (lead); Investigation (equal); Validation (equal); Writing – original draft (lead). **Zhenzhen Cui:** Investigation (equal). **Meihong Ning:** Investigation (equal). **Yu Chen:** Investigation (equal). **Zhengjun Wu:** Funding acquisition (equal); Methodology (equal); Resources (equal). **Huayuan Huang:** Conceptualization (equal); Funding acquisition (equal); Project administration (equal); Supervision (equal); Visualization (equal); Writing – review & editing (equal).

### OPEN RESEARCH BADGES

All data are available in the Dryad repository, and the link to the data is https://doi.org/10.5061/dryad.zs7h44j9t.

## Supporting information

Appendix S1‐S3

## Data Availability

The raw sequencing data from the current study are available in the Dryad repository at https://doi.org/10.5061/dryad.zs7h44j9t.
