# Supplementary material for: Variation in the intestinal microbiota at different developmental stages of Hynobius maoershanensis
Source: Ecol Evol. 2022 Mar 18;12(3):e8712. doi: 10.1002/ece3.8712 (PMC8931708; doi:10.1002/ece3.8712)
Supplement: Supplementary file 1 — Appendix S1‐S3 [file ECE3-12-e8712-s001.docx]

**Appendix**

**Appendix S1** The proportion differences in the relative abundance of gut microbiota community of *H. maoershanensis* in different developmental stages (at the phylum level)

|  | **Relative abundance （%）** | | | **N = 73** | |
| --- | --- | --- | --- | --- | --- |
| **Species name** | **adult** | **hindlimb bud stage** | **forelimb bud stage** | **Pvalue** | **Corrected pvalue** |
| Firmicutes | 7.27±11.67 | 58.9±28.41 | 52.19±31.03 | 0.000 | 0.000 |
| Proteobacteria | 44.17±21.84 | 21.77±21.32 | 30.31±27.74 | 0.007 | 0.057 |
| Bacteroidetes | 39.69±21.4 | 9.38±16.37 | 1.99±2.7 | 0.000 | 0.000 |
| Fusobacteria | 0.01±0.02 | 1.99±3.29 | 9.73±13.59 | 0.000 | 0.000 |
| Actinobacteria | 2.63±5.25 | 3.31±4.46 | 4.58±8.35 | 0.667 | 0.752 |
| Deferribacteres | 3.47±9.32 | 0.26±0.53 | 0.21±0.72 | 0.004 | 0.042 |
| Acidobacteria | 0.42±1.25 | 1.6±4.43 | 0.16±0.36 | 0.385 | 0.611 |
| Verrucomicrobia | 0.31±0.58 | 0.79±1.4 | 0.03±0.06 | 0.001 | 0.007 |
| unclassified_k__norank_d__Bacteria | 0.48±0.78 | 0.24±0.37 | 0.32±0.42 | 0.447 | 0.611 |
| Tenericutes | 0.05±0.18 | 0.65±2.13 | 0.03±0.06 | 0.173 | 0.466 |
| Cyanobacteria | 0.12±0.2 | 0.31±0.43 | 0.14±0.23 | 0.309 | 0.556 |
| unclassified_d__Unclassified | 0.33±0.72 | 0.2±0.57 | 0.01±0.02 | 0.000 | 0.004 |
| Chloroflexi | 0.2±0.69 | 0.24±0.32 | 0.07±0.13 | 0.115 | 0.368 |
| Spirochaetes | 0.35±1.15 | 0.02±0.07 | 0.01±0.01 | 0.119 | 0.368 |
| Planctomycetes | 0.05±0.15 | 0.05±0.09 | 0.05±0.11 | 0.028 | 0.159 |
| TM7 | 0.08±0.25 | 0.04±0.06 | 0.02±0.03 | 0.012 | 0.085 |
| [Thermi] | 0.01±0.03 | 0.03±0.07 | 0.05±0.24 | 0.258 | 0.556 |
| Chlorobi | 0.05±0.26 | 0.01±0.04 | 0.01±0.03 | 0.824 | 0.866 |
| OD1 | 0.03±0.16 | 0.02±0.06 | 0.02±0.05 | 0.529 | 0.619 |
| Nitrospirae | 0.04±0.22 | 0.02±0.03 | 0.00±0.01 | 0.048 | 0.231 |
| Chlamydiae | 0.03±0.07 | 0.02±0.03 | 0.01±0.04 | 0.542 | 0.622 |
| Gemmatimonadetes | 0.02±0.05 | 0.02±0.04 | 0.01±0.04 | 0.112 | 0.368 |
| Elusimicrobia | 0.03±0.07 | 0.01±0.02 | 0.01±0.02 | 0.399 | 0.611 |
| Lentisphaerae | 0.04±0.15 | 0.00±0.00 | 0.00±0.00 | 0.006 | 0.051 |
| WPS-2 | 0.01±0.02 | 0.02±0.03 | 0.01±0.03 | 0.031 | 0.161 |
| GN02 | 0.01±0.07 | 0.02±0.05 | 0.00±0.01 | 0.952 | 0.952 |
| WS3 | 0.01±0.05 | 0.02±0.06 | 0.00±0.02 | 0.246 | 0.556 |
| TM6 | 0.01±0.04 | 0.01±0.02 | 0.00±0.01 | 0.022 | 0.134 |
| NC10 | 0.02±0.06 | 0.00±0.01 | 0.00±0.02 | 0.891 | 0.909 |
| TPD-58 | 0.02±0.06 | 0.00±0.00 | 0.00±0.00 | 0.227 | 0.556 |
| WS5 | 0.01±0.05 | 0.00±0.00 | 000±0.01 | 0.134 | 0.397 |
| Armatimonadetes | 0.00±0.02 | 0.01±0.02 | 0.00±0.00 | 0.341 | 0.556 |
| OP3 | 0.01±0.03 | 0.00±0.00 | 0.00±0.01 | 0.894 | 0.909 |
| SR1 | 0.01±0.04 | 0.00±0.01 | 0.00±0.00 | 0.328 | 0.556 |
| AD3 | 0.00±0.01 | 0.01±0.02 | 0.00±0.00 | 0.286 | 0.556 |
| GAL15 | 0.01±0.03 | 0.00±0.00 | 0.00±0.00 | 0.110 | 0.368 |

**Appendix S2.** The proportion differences in the relative abundance of gut microbiota community of *H. maoershanensis* in different developmental stages (at the family level)

|  | **Relative abundance （%）** | | | **N = 73** | |
| --- | --- | --- | --- | --- | --- |
| **Species name** | **adult** | **hindlimb bud stage** | **forelimb bud stage** | **Pvalue** | **Corrected pvalue** |
| Peptostreptococcaceae | 0.01±0.01 | 33.66±34.21 | 10.27±13.62 | 0.000 | 0.000 |
| Oxalobacteraceae | 28.28±18.9 | 0.70±1.12 | 5.58±13.28 | 0.000 | 0.000 |
| Flavobacteriaceae | 30.02±23.83 | 0.08±0.19 | 0.25±0.96 | 0.000 | 0.000 |
| Clostridiaceae | 0.03±0.07 | 9.97±12.04 | 19.31±16.58 | 0.000 | 0.000 |
| Enterobacteriaceae | 1.54±1.24 | 9.12±14.49 | 11.35±18.71 | 0.632 | 0.720 |
| Lachnospiraceae | 1.05±2.49 | 3.9±7.06 | 8.41±10.98 | 0.000 | 0.001 |
| Pseudomonadaceae | 3.78±10.7 | 0.28±0.5 | 8.13±20.07 | 0.285 | 0.568 |
| Fusobacteriaceae | 0.01±0.02 | 1.99±3.29 | 9.72±13.59 | 0.000 | 0.000 |
| Ruminococcaceae | 2.60±4.29 | 3.70±4.00 | 1.57±2.34 | 0.111 | 0.350 |
| Rikenellaceae | 3.62±6.92 | 3.77±11.27 | 0.33±1.10 | 0.000 | 0.005 |
| Veillonellaceae | 0.18±0.27 | 1.14±2.48 | 5.52±12.29 | 0.589 | 0.694 |
| Comamonadaceae | 0.22±0.6 | 4.08±12.38 | 1.75±2.64 | 0.000 | 0.001 |
| Nocardiaceae | 1.97±4.28 | 0.05±0.09 | 3.88±7.75 | 0.011 | 0.090 |
| norank_o__Clostridiales | 1.96±4.93 | 0.41±0.88 | 2.50±4.59 | 0.583 | 0.691 |
| Deferribacteraceae | 3.47±9.32 | 0.26±0.53 | 0.21±0.72 | 0.004 | 0.046 |
| Porphyromonadaceae | 0.97±1.56 | 1.93±3.18 | 0.42±1.21 | 0.010 | 0.085 |
| Bacteroidaceae_o__Bacteroidales | 1.99±4.17 | 0.87±1.52 | 0.26±0.73 | 0.015 | 0.115 |
| norank_o__Rickettsiales | 2.59±3.64 | 0.39±0.91 | 0.01±0.04 | 0.000 | 0.000 |
| unclassified_o__Clostridiales | 0.44±0.68 | 1.82±2.13 | 0.69±1.51 | 0.001 | 0.021 |
| Turicibacteraceae | 0.00±0.00 | 1.71±3.03 | 0.97±3.1 | 0.000 | 0.000 |
| unclassified_o__Bacteroidales_c__Bacteroidia | 1.16±2.27 | 0.93±1.43 | 0.18±0.77 | 0.000 | 0.008 |
| Alcaligenaceae | 2.23±2.41 | 0.01±0.01 | 0.01±0.04 | 0.000 | 0.000 |
| Desulfovibrionaceae | 0.3±0.62 | 1.25±2.05 | 0.17±0.63 | 0.003 | 0.035 |
| Burkholderiaceae | 0.18±0.45 | 0.61±1.68 | 0.81±2.15 | 0.748 | 0.829 |
| Erysipelotrichaceae | 0.18±0.55 | 0.59±0.78 | 0.61±1.63 | 0.233 | 0.537 |
| Acidobacteriaceae | 0.01±0.05 | 1.28±4.35 | 0.00±0.01 | 0.276 | 0.568 |
| Moraxellaceae | 0.13±0.38 | 0.69±1.76 | 0.38±1.07 | 0.957 | 0.975 |
| Bacillaceae | 0.05±0.17 | 0.17±0.21 | 0.92±2.47 | 0.002 | 0.030 |
| [Odoribacteraceae] | 0.79±1.51 | 0.26±0.37 | 0.00±0.00 | 0.000 | 0.000 |
| unclassified_k_norank_d__Bacteria | 0.48±0.78 | 0.24±0.37 | 0.32±0.42 | 0.447 | 0.624 |
| unclassified_c__Betaproteobacteria | 0.91±1.50 | 0.05±0.12 | 0.04±0.07 | 0.000 | 0.000 |
| Rickettsiaceae | 0.96±1.14 | 0.03±0.10 | 0.00±0.01 | 0.000 | 0.000 |
| norank_o__Bacteroidales | 0.83±1.70 | 0.11±0.21 | 0.04±0.10 | 0.003 | 0.037 |
| Microbacteriaceae | 0.03±0.08 | 0.79±1.58 | 0.13±0.43 | 0.119 | 0.370 |
| Verrucomicrobiaceae | 0.16±0.30 | 0.72±1.43 | 0.01±0.03 | 0.002 | 0.033 |
| Sphingobacteriaceae | 0.02±0.04 | 0.69±1.87 | 0.04±0.12 | 0.045 | 0.244 |
| Rhodospirillaceae | 0.35±0.78 | 0.35±0.91 | 0.04±0.08 | 0.223 | 0.537 |
| Methylocystaceae | 0.30±0.93 | 0.36±0.56 | 0.07±0.13 | 0.001 | 0.012 |
| Sphingomonadaceae | 0.14±0.33 | 0.35±0.59 | 0.20±0.49 | 0.564 | 0.674 |
| Aeromonadaceae | 0.03±0.08 | 0.03±0.07 | 0.60±2.21 | 0.983 | 0.985 |
| Enterococcaceae | 0.07±0.25 | 0.52±0.88 | 0.07±0.16 | 0.000 | 0.009 |
| [Tissierellaceae] | 0.13±0.32 | 0.02±0.03 | 0.42±1.05 | 0.867 | 0.900 |
| **Appendix S2 (Continued)** |  |  |  |  |  |
|  | **Relative abundance （%）** | | | **N = 73** | |
| **Species name** | **adult** | **hindlimb bud stage** | **forelimb bud stage** | **Pvalue** | **Corrected pvalue** |
| Caulobacteraceae | 0.07±0.21 | 0.31±0.46 | 0.16±0.36 | 0.396 | 0.624 |
| Rhodocyclaceae | 0.26±0.82 | 0.20±0.45 | 0.08±0.11 | 0.003 | 0.037 |
| unclassified_d__Unclassified | 0.33±0.72 | 0.20±0.57 | 0.01±0.02 | 0.000 | 0.006 |
| Staphylococcaceae | 0.06±0.23 | 0.36±1.03 | 0.10±0.22 | 0.127 | 0.377 |
| norank_o__Entomoplasmatales | 0.00±0.00 | 0.48±1.65 | 0.01±0.04 | 0.125 | 0.376 |
| Micrococcaceae | 0.03±0.06 | 0.35±0.69 | 0.09±0.29 | 0.032 | 0.185 |
| Xanthomonadaceae | 0.05±0.12 | 0.32±0.60 | 0.08±0.15 | 0.272 | 0.568 |
| Chitinophagaceae | 0.11±0.20 | 0.24±0.50 | 0.08±0.21 | 0.129 | 0.381 |
| [Mogibacteriaceae] | 0.06±0.15 | 0.32±0.77 | 0.03±0.06 | 0.042 | 0.233 |
| Hyphomicrobiaceae | 0.20±0.65 | 0.17±0.23 | 0.03±0.05 | 0.011 | 0.088 |
| Spirochaetaceae | 0.35±1.15 | 0.02±0.06 | 0.00±0.01 | 0.018 | 0.130 |
| Cellulomonadaceae | 0.00±0.01 | 0.34±0.78 | 0.01±0.03 | 0.000 | 0.002 |
| [Weeksellaceae] | 0.05±0.13 | 0.16±0.34 | 0.15±0.43 | 0.521 | 0.636 |
| Paenibacillaceae | 0.03±0.07 | 0.09±0.16 | 0.21±0.40 | 0.014 | 0.107 |
| unclassified_p__Proteobacteria | 0.03±0.05 | 0.28±0.94 | 0.00±0.01 | 0.000 | 0.005 |
| Planococcaceae | 0.05±0.13 | 0.04±0.07 | 0.23±0.65 | 0.119 | 0.370 |
| S24-7 | 0.03±0.07 | 0.24±0.81 | 0.04±0.10 | 0.017 | 0.130 |
| Crenotrichaceae | 0.13±0.40 | 0.14±0.44 | 0.03±0.10 | 0.893 | 0.922 |
| Mycobacteriaceae | 0.03±0.10 | 0.23±0.32 | 0.03±0.06 | 0.000 | 0.001 |
| Frankiaceae | 0.00±0.00 | 0.29±0.99 | 0.00±0.00 | 0.000 | 0.001 |
| unclassified_c__Gammaproteobacteria | 0.07±0.20 | 0.17±0.35 | 0.05±0.08 | 0.005 | 0.053 |
| Procabacteriaceae | 0.27±0.35 | 0.00±0.01 | 0.01±0.01 | 0.000 | 0.000 |
| unclassified_c_Alphaproteobacteria | 0.00±0.01 | 0.25±0.80 | 0.01±0.03 | 0.234 | 0.537 |
| Myxococcaceae | 0.13±0.41 | 0.08±0.18 | 0.05±0.09 | 0.524 | 0.637 |
| Bradyrhizobiaceae | 0.06±0.18 | 0.13±0.24 | 0.05±0.10 | 0.005 | 0.051 |
| Koribacteraceae | 0.10±0.36 | 0.10±0.24 | 0.03±0.08 | 0.035 | 0.194 |
| Acetobacteraceae | 0.07±0.23 | 0.13±0.28 | 0.03±0.05 | 0.076 | 0.281 |
| norank_o__Actinomycetales | 0.02±0.08 | 0.19±0.58 | 0.01±0.03 | 0.317 | 0.568 |
| norank_o__SC-I-84 | 0.17±0.54 | 0.03±0.05 | 0.01±0.02 | 0.062 | 0.281 |
| unclassified_p__Firmicutes | 0.01±0.03 | 0.17±0.22 | 0.02±0.05 | 0.013 | 0.104 |
| norank_c__OPB41 | 0.09±0.27 | 0.08±0.13 | 0.04±0.07 | 0.446 | 0.624 |
| Xanthobacteraceae | 0.00±0.00 | 0.19±0.65 | 0.00±0.01 | 0.290 | 0.568 |
| Conexibacteraceae | 0.01±0.02 | 0.18±0.57 | 0.01±0.01 | 0.120 | 0.370 |
| Coriobacteriaceae | 0.04±0.05 | 0.13±0.35 | 0.02±0.04 | 0.003 | 0.038 |
| Sinobacteraceae | 0.04±0.16 | 0.12±0.26 | 0.01±0.04 | 0.434 | 0.624 |
| Lactobacillaceae | 0.08±0.23 | 0.01±0.03 | 0.08±0.25 | 0.229 | 0.537 |
| Neisseriaceae | 0.10±0.36 | 0.03±0.05 | 0.04±0.08 | 0.738 | 0.823 |
| norank_c__Ellin6529 | 0.07±0.23 | 0.07±0.10 | 0.02±0.06 | 0.021 | 0.144 |
| Rhodobacteraceae | 0.02±0.05 | 0.08±0.14 | 0.05±0.15 | 0.319 | 0.568 |
| norank_o__Streptophyta | 0.01±0.05 | 0.07±0.12 | 0.05±0.18 | 0.029 | 0.172 |
| Geobacteraceae | 0.06±0.19 | 0.06±0.18 | 0.02±0.04 | 0.792 | 0.861 |
| norank_o__Stramenopiles | 0.00±0.02 | 0.09±0.21 | 0.03±0.06 | 0.000 | 0.008 |
| Propionibacteriaceae | 0.04±0.13 | 0.02±0.05 | 0.06±0.14 | 0.226 | 0.537 |
| norank_o__iii1-15 | 0.07±0.27 | 0.03±0.08 | 0.02±0.08 | 0.295 | 0.568 |
| **Appendix S2 (Continued)** |  |  |  |  |  |
|  | **Relative abundance （%）** | | | **N = 73** | |
| **Species name** | **adult** | **hindlimb bud stage** | **forelimb bud stage** | **Pvalue** | **Corrected pvalue** |
| norank_o__Acidimicrobiales | 0.06±0.16 | 0.05±0.08 | 0.01±0.03 | 0.023 | 0.153 |
| Corynebacteriaceae | 0.02±0.07 | 0.06±0.12 | 0.05±0.13 | 0.028 | 0.172 |
| norank_o__YS2 | 0.09±0.18 | 0.03±0.05 | 0.00±0.01 | 0.005 | 0.055 |
| norank_o__Rhizobiales | 0.02±0.06 | 0.08±0.16 | 0.01±0.03 | 0.169 | 0.469 |
| Cytophagaceae | 0.01±0.06 | 0.04±0.11 | 0.06±0.16 | 0.029 | 0.172 |
| Prevotellaceae | 0.03±0.06 | 0.01±0.02 | 0.08±0.26 | 0.055 | 0.275 |
| norank_o__Solibacterales | 0.08±0.26 | 0.03±0.07 | 0.01±0.02 | 0.283 | 0.568 |
| Christensenellaceae | 0.04±0.06 | 0.02±0.03 | 0.05±0.14 | 0.751 | 0.829 |
| Streptococcaceae | 0.01±0.02 | 0.05±0.09 | 0.05±0.17 | 0.241 | 0.552 |
| norank_o__Myxococcales | 0.02±0.06 | 0.07±0.19 | 0.02±0.03 | 0.196 | 0.519 |
| Brevibacteriaceae | 0.03±0.13 | 0.06±0.15 | 0.01±0.03 | 0.001 | 0.014 |
| Holophagaceae | 0.03±0.12 | 0.02±0.07 | 0.05±0.09 | 0.437 | 0.624 |
| [Exiguobacteraceae] | 0.05±0.22 | 0.02±0.06 | 0.03±0.10 | 0.930 | 0.949 |
| Nakamurellaceae | 0.00±0.00 | 0.10±0.34 | 0.00±0.00 | 0.316 | 0.568 |
| unclassified_o_Entomoplasmatales | 0.00±0.00 | 0.10±0.33 | 0.00±0.00 | 0.079 | 0.281 |
| unclassified_o__Burkholderiales | 0.01±0.04 | 0.01±0.02 | 0.08±0.29 | 0.358 | 0.600 |
| Nocardioidaceae | 0.01±0.03 | 0.06±0.09 | 0.02±0.04 | 0.003 | 0.035 |
| unclassified_o__Bacillales | 0.00±0.00 | 0.07±0.17 | 0.02±0.07 | 0.120 | 0.370 |
| norank_c__Actinobacteria | 0.05±0.19 | 0.02±0.02 | 0.01±0.02 | 0.084 | 0.296 |
| Deinococcaceae | 0.00±0.01 | 0.02±0.05 | 0.05±0.24 | 0.123 | 0.373 |
| Rhizobiaceae | 0.01±0.03 | 0.03±0.06 | 0.04±0.10 | 0.801 | 0.864 |
| norank_o__Ellin6513 | 0.02±0.07 | 0.03±0.07 | 0.02±0.05 | 0.492 | 0.624 |
| unclassified_o__Actinomycetales | 0.01±0.03 | 0.05±0.06 | 0.01±0.02 | 0.003 | 0.037 |
| norank_c__Betaproteobacteria | 0.05±0.23 | 0.01±0.03 | 0.01±0.04 | 0.528 | 0.640 |
| norank_c__SC3 | 0.05±0.17 | 0.01±0.04 | 0.00±0.01 | 0.841 | 0.880 |
| Carnobacteriaceae | 0.01±0.04 | 0.01±0.01 | 0.05±0.20 | 0.070 | 0.281 |
| Alicyclobacillaceae | 0.06±0.33 | 0.00±0.00 | 0.00±0.00 | 0.450 | 0.624 |
| norank_o__Ellin329 | 0.01±0.03 | 0.05±0.16 | 0.00±0.00 | 0.171 | 0.471 |
| Intrasporangiaceae | 0.01±0.02 | 0.03±0.04 | 0.03±0.06 | 0.126 | 0.376 |
| Actinomycetaceae | 0.01±0.03 | 0.01±0.03 | 0.04±0.14 | 0.263 | 0.568 |
| unclassified_c__Clostridia | 0.00±0.00 | 0.02±0.05 | 0.04±0.09 | 0.149 | 0.423 |
| Ellin515 | 0.05±0.18 | 0.00±0.01 | 0.00±0.00 | 0.962 | 0.977 |
| Isosphaeraceae | 0.02±0.07 | 0.03±0.05 | 0.01±0.03 | 0.030 | 0.176 |
| Solibacteraceae | 0.02±0.08 | 0.03±0.09 | 0.00±0.01 | 0.134 | 0.390 |
| Dermabacteraceae | 0.01±0.03 | 0.04±0.10 | 0.01±0.04 | 0.021 | 0.144 |
| Campylobacteraceae | 0.00±0.00 | 0.05±0.13 | 0.00±0.00 | 0.010 | 0.084 |
| Bacteriovoracaceae | 0.00±0.01 | 0.05±0.09 | 0.00±0.01 | 0.023 | 0.153 |
| Coxiellaceae | 0.04±0.16 | 0.01±0.03 | 0.00±0.01 | 0.397 | 0.624 |
| auto67_4W | 0.03±0.1 | 0.02±0.08 | 0.00±0.01 | 0.756 | 0.830 |
| Bifidobacteriaceae | 0.02±0.03 | 0.00±0.00 | 0.03±0.12 | 0.220 | 0.537 |
| unclassified_p__Bacteroidetes | 0.03±0.09 | 0.01±0.01 | 0.02±0.06 | 0.354 | 0.595 |
| Phyllobacteriaceae | 0.01±0.03 | 0.01±0.02 | 0.03±0.08 | 0.182 | 0.487 |
| norank_o__CCU21 | 0.02±0.08 | 0.02±0.06 | 0.01±0.05 | 0.785 | 0.858 |
| norank_o__Sphingobacteriales | 0.02±0.08 | 0.02±0.04 | 0.01±0.02 | 0.607 | 0.709 |
| EB1017 | 0.02±0.06 | 0.02±0.04 | 0.01±0.03 | 0.028 | 0.172 |
| **Appendix S2 (Continued)** |  |  |  |  |  |
|  | **Relative abundance （%）** | | | **N = 73** | |
| **Species name** | **adult** | **hindlimb bud stage** | **forelimb bud stage** | **Pvalue** | **Corrected pvalue** |
| norank_o__Gaiellales | 0.03±0.08 | 0.01±0.02 | 0.01±0.02 | 0.441 | 0.624 |
| Methylophilaceae | 0.02±0.09 | 0.01±0.03 | 0.01±0.03 | 0.564 | 0.674 |
| norank_c__BD7-11 | 0.01±0.03 | 0.00±0.01 | 0.03±0.09 | 0.264 | 0.568 |
| Beijerinckiaceae | 0.01±0.04 | 0.02±0.03 | 0.01±0.03 | 0.000 | 0.008 |
| Thermogemmatisporaceae | 0.01±0.05 | 0.03±0.05 | 0.00±0.00 | 0.069 | 0.281 |
| norank_o__Burkholderiales | 0.00±0.01 | 0.03±0.12 | 0.01±0.02 | 0.467 | 0.624 |
| Mycoplasmataceae | 0.04±0.18 | 0.00±0.00 | 0.00±0.00 | 0.000 | 0.008 |
| Eubacteriaceae | 0.02±0.09 | 0.02±0.03 | 0.00±0.00 | 0.000 | 0.000 |
| norank_c__TM7-1 | 0.02±0.07 | 0.01±0.02 | 0.01±0.01 | 0.015 | 0.117 |
| Desulfobacteraceae | 0.03±0.14 | 0.00±0.01 | 0.01±0.03 | 0.966 | 0.977 |
| Victivallaceae | 0.04±0.15 | 0.00±0.00 | 0.00±0.00 | 0.006 | 0.055 |
| norank_o__Chlorophyta | 0.00±0.00 | 0.04±0.08 | 0.01±0.01 | 0.002 | 0.028 |
| Peptococcaceae | 0.02±0.03 | 0.02±0.04 | 0.00±0.00 | 0.006 | 0.058 |
| Syntrophobacteraceae | 0.03±0.10 | 0.01±0.02 | 0.00±0.02 | 0.862 | 0.897 |
| unclassified_o_Solirubrobacterales | 0.01±0.03 | 0.03±0.03 | 0.00±0.01 | 0.001 | 0.018 |
| Legionellaceae | 0.01±0.02 | 0.00±0.01 | 0.03±0.13 | 0.332 | 0.568 |
| Trebouxiophyceae | 0.00±0.00 | 0.04±0.13 | 0.00±0.00 | 0.000 | 0.000 |
| norank_o__[Pedosphaerales] | 0.03±0.12 | 0.01±0.02 | 0.00±0.01 | 0.968 | 0.977 |
| Syntrophaceae | 0.02±0.1 | 0.01±0.02 | 0.00±0.01 | 0.143 | 0.408 |
| Bdellovibrionaceae | 0.01±0.03 | 0.02±0.03 | 0.01±0.03 | 0.217 | 0.537 |
| unclassified_p__Tenericutes | 0.00±0.02 | 0.03±0.05 | 0.00±0.01 | 0.001 | 0.016 |
| norank_o__OPB54 | 0.01±0.03 | 0.02±0.04 | 0.01±0.01 | 0.034 | 0.193 |
| Anaerolinaceae | 0.02±0.07 | 0.01±0.03 | 0.00±0.01 | 0.337 | 0.572 |
| Gaiellaceae | 0.01±0.05 | 0.01±0.03 | 0.01±0.03 | 0.499 | 0.624 |
| FW | 0.03±0.18 | 0.00±0.00 | 0.00±0.01 | 0.983 | 0.985 |
| norank_p__WPS-2 | 0.01±0.02 | 0.02±0.03 | 0.01±0.03 | 0.031 | 0.181 |
| norank_o__Ellin6067 | 0.03±0.11 | 0.00±0.02 | 0.00±0.01 | 0.842 | 0.880 |
| Methylococcaceae | 0.01±0.04 | 0.02±0.06 | 0.01±0.02 | 0.223 | 0.537 |
| Methylobacteriaceae | 0.00±0.01 | 0.02±0.03 | 0.01±0.04 | 0.012 | 0.096 |
| norank_c__Alphaproteobacteria | 0.00±0.02 | 0.02±0.05 | 0.01±0.03 | 0.027 | 0.172 |
| Ktedonobacteraceae | 0.00±0.02 | 0.02±0.05 | 0.00±0.00 | 0.001 | 0.017 |
| Leuconostocaceae | 0.02±0.10 | 0.00±0.00 | 0.01±0.03 | 0.209 | 0.535 |
| norank_o__SJA-36 | 0.02±0.08 | 0.00±0.01 | 0.00±0.01 | 0.790 | 0.861 |
| Phormidiaceae | 0.00±0.00 | 0.00±0.00 | 0.03±0.12 | 0.004 | 0.047 |
| norank_o__RF32 | 0.02±0.05 | 0.01±0.02 | 0.00±0.00 | 0.007 | 0.068 |
| Rhabdochlamydiaceae | 0.01±0.04 | 0.00±0.01 | 0.01±0.03 | 0.855 | 0.891 |
| Halomonadaceae | 0.00±0.01 | 0.01±0.01 | 0.02±0.05 | 0.792 | 0.861 |
| Chromatiaceae | 0.00±0.01 | 0.02±0.04 | 0.01±0.03 | 0.512 | 0.627 |
| [Chthoniobacteraceae] | 0.01±0.04 | 0.01±0.02 | 0.00±0.01 | 0.024 | 0.158 |
| Pseudonocardiaceae | 0.01±0.02 | 0.02±0.03 | 0.00±0.01 | 0.046 | 0.249 |
| Aerococcaceae | 0.01±0.02 | 0.02±0.05 | 0.00±0.00 | 0.103 | 0.339 |
| unclassified_c__Mollicutes | 0.00±0.00 | 0.02±0.08 | 0.00±0.00 | 0.099 | 0.332 |
| Gemmataceae | 0.01±0.04 | 0.01±0.02 | 0.00±0.01 | 0.231 | 0.537 |
| oc28 | 0.00±0.03 | 0.02±0.05 | 0.00±0.00 | 0.092 | 0.314 |
| 0319-6G20 | 0.00±0.01 | 0.01±0.01 | 0.01±0.04 | 0.438 | 0.624 |
| **Appendix S2 (Continued)** |  |  |  |  |  |
|  | **Relative abundance （%）** | | | **N = 73** | |
| **Species name** | **adult** | **hindlimb bud stage** | **forelimb bud stage** | **Pvalue** | **Corrected pvalue** |
| norank_o__Thiobacterales | 0.00±0.00 | 0.02±0.07 | 0.00±0.01 | 0.283 | 0.568 |
| norank_c__ZB2 | 0.01±0.04 | 0.01±0.02 | 0.01±0.03 | 0.674 | 0.759 |
| Dehalobacteriaceae | 0.01±0.03 | 0.00±0.01 | 0.01±0.02 | 0.252 | 0.553 |
| norank_o__MIZ46 | 0.01±0.02 | 0.01±0.03 | 0.01±0.02 | 0.172 | 0.471 |
| unclassified_o__Rhizobiales | 0.01±0.03 | 0.01±0.03 | 0.00±0.00 | 0.178 | 0.480 |
| norank_o__MLE1-12 | 0.01±0.02 | 0.01±0.04 | 0.01±0.02 | 0.267 | 0.568 |
| Elusimicrobiaceae | 0.02±0.07 | 0.00±0.00 | 0.00±0.00 | 0.019 | 0.133 |
| PRR-10 | 0.01±0.04 | 0.01±0.05 | 0.00±0.01 | 0.405 | 0.624 |
| norank_c__CK-1C4-19 | 0.00±0.00 | 0.01±0.03 | 0.01±0.02 | 0.002 | 0.032 |
| norank_o__JH-WHS47 | 0.02±0.06 | 0.00±0.01 | 0.00±0.02 | 0.891 | 0.922 |
| Kineosporiaceae | 0.00±0.00 | 0.02±0.05 | 0.00±0.02 | 0.027 | 0.172 |
| Ignavibacteriaceae | 0.02±0.09 | 0.00±0.01 | 0.00±0.01 | 0.331 | 0.568 |
| norank_p__OD1 | 0.01±0.06 | 0.00±0.01 | 0.01±0.02 | 0.139 | 0.399 |
| norank_o__JG30-KF-CM45 | 0.00±0.01 | 0.01±0.02 | 0.01±0.02 | 0.707 | 0.795 |
| norank_c__BD1-5 | 0.00±0.00 | 0.02±0.05 | 0.00±0.01 | 0.322 | 0.568 |
| norank_c__Gemm-1 | 0.01±0.03 | 0.01±0.03 | 0.00±0.00 | 0.018 | 0.133 |
| Brucellaceae | 0.01±0.02 | 0.00±0.00 | 0.01±0.03 | 0.364 | 0.608 |
| unclassified_o__Myxococcales | 0.01±0.03 | 0.01±0.02 | 0.00±0.01 | 0.653 | 0.742 |
| Opitutaceae | 0.00±0.02 | 0.01±0.03 | 0.00±0.01 | 0.248 | 0.553 |
| C111 | 0.00±0.01 | 0.00±0.01 | 0.01±0.05 | 0.550 | 0.662 |
| norank_p__TPD-58 | 0.02±0.06 | 0.00±0.00 | 0.00±0.00 | 0.227 | 0.537 |
| unclassified_o__Sphingomonadales | 0.00±0.00 | 0.02±0.04 | 0.00±0.00 | 0.152 | 0.429 |
| Dietziaceae | 0.00±0.00 | 0.01±0.03 | 0.00±0.01 | 0.020 | 0.136 |
| norank_o__SJA-15 | 0.01±0.03 | 0.01±0.01 | 0.00±0.01 | 0.271 | 0.568 |
| Micromonosporaceae | 0.00±0.01 | 0.01±0.01 | 0.01±0.04 | 0.062 | 0.281 |
| norank_o__envOPS12 | 0.01±0.08 | 0.00±0.00 | 0.00±0.01 | 0.751 | 0.829 |
| Dermacoccaceae | 0.00±0.01 | 0.00±0.00 | 0.01±0.03 | 0.385 | 0.624 |
| unclassified_o__[Pedosphaerales] | 0.01±0.05 | 0.00±0.01 | 0.00±0.00 | 0.840 | 0.880 |
| Ellin6075 | 0.00±0.02 | 0.01±0.02 | 0.00±0.00 | 0.982 | 0.985 |
| norank_o__Elusimicrobiales | 0.00±0.01 | 0.01±0.01 | 0.01±0.02 | 0.408 | 0.624 |
| norank_c__ABY1 | 0.01±0.05 | 0.01±0.02 | 0.00±0.00 | 0.611 | 0.709 |
| [Thermodesulfovibrionaceae] | 0.01±0.02 | 0.01±0.02 | 0.00±0.00 | 0.419 | 0.624 |
| norank_o__BD7-3 | 0.01±0.02 | 0.00±0.01 | 0.01±0.02 | 0.922 | 0.943 |
| Vibrionaceae | 0.01±0.04 | 0.00±0.00 | 0.00±0.01 | 0.158 | 0.445 |
| norank_p__WS5 | 0.01±0.05 | 0.00±0.00 | 0.00±0.01 | 0.134 | 0.390 |
| unclassified_o__Legionellales | 0.00±0.00 | 0.01±0.03 | 0.00±0.01 | 0.062 | 0.281 |
| norank_o__Spirobacillales | 0.00±0.01 | 0.01±0.02 | 0.00±0.00 | 0.006 | 0.055 |
| unclassified_c__Anaerolineae | 0.01±0.04 | 0.00±0.01 | 0.00±0.01 | 0.906 | 0.933 |
| norank_c__SJA-4 | 0.01±0.03 | 0.01±0.01 | 0.00±0.00 | 0.003 | 0.035 |
| Streptomycetaceae | 0.01±0.01 | 0.00±0.00 | 0.00±0.02 | 0.138 | 0.399 |
| norank_c__TM7-3 | 0.00±0.01 | 0.01±0.02 | 0.01±0.02 | 0.090 | 0.311 |
| unclassified_c__Actinobacteria | 0.00±0.00 | 0.01±0.03 | 0.00±0.00 | 0.227 | 0.537 |
| norank_c__ML635J-21 | 0.00±0.01 | 0.01±0.03 | 0.00±0.00 | 0.425 | 0.624 |
| Gracilibacteraceae | 0.00±0.00 | 0.01±0.03 | 0.00±0.00 | 0.000 | 0.008 |
| norank_o__[Entotheonellales] | 0.01±0.06 | 0.00±0.00 | 0.00±0.01 | 0.618 | 0.711 |
| **Appendix S2 (Continued)** |  |  |  |  |  |
|  | **Relative abundance （%）** | | | **N = 73** | |
| **Species name** | **adult** | **hindlimb bud stage** | **forelimb bud stage** | **Pvalue** | **Corrected pvalue** |
| norank_c__Deltaproteobacteria | 0.01±0.04 | 0.00±0.01 | 0.00±0.00 | 0.593 | 0.697 |
| norank_o__S0208 | 0.00±0.00 | 0.01±0.04 | 0.00±0.00 | 0.079 | 0.281 |
| norank_o__Sva0725 | 0.00±0.00 | 0.01±0.04 | 0.00±0.00 | 0.079 | 0.281 |
| norank_c__OPB56 | 0.00±0.02 | 0.01±0.02 | 0.00±0.01 | 0.968 | 0.977 |
| unclassified_c_Deltaproteobacteria | 0.00±0.01 | 0.01±0.01 | 0.00±0.00 | 0.029 | 0.172 |
| norank_o__A31 | 0.01±0.05 | 0.00±0.01 | 0.00±0.00 | 0.968 | 0.977 |
| Desulfobulbaceae | 0.00±0.01 | 0.01±0.03 | 0.00±0.01 | 0.398 | 0.624 |
| Patulibacteraceae | 0.00±0.00 | 0.01±0.03 | 0.00±0.00 | 0.000 | 0.000 |
| unclassified_o__Chlamydiales | 0.01±0.03 | 0.00±0.01 | 0.00±0.00 | 0.553 | 0.664 |
| Saprospiraceae | 0.00±0.02 | 0.00±0.01 | 0.00±0.01 | 0.486 | 0.624 |
| norank_o__0319-7L14 | 0.00±0.00 | 0.01±0.03 | 0.00±0.00 | 0.092 | 0.314 |
| [Paraprevotellaceae] | 0.00±0.01 | 0.00±0.00 | 0.01±0.02 | 0.584 | 0.691 |
| Symbiobacteriaceae | 0.00±0.01 | 0.01±0.02 | 0.00±0.01 | 0.459 | 0.624 |
| norank_p__SR1 | 0.01±0.04 | 0.00±0.01 | 0.00±0.00 | 0.328 | 0.568 |
| Thermaceae | 0.00±0.01 | 0.01±0.02 | 0.00±0.00 | 0.057 | 0.279 |
| Polyangiaceae | 0.00±0.00 | 0.00±0.00 | 0.01±0.03 | 0.619 | 0.711 |
| Leptotrichiaceae | 0.00±0.00 | 0.01±0.02 | 0.00±0.01 | 0.616 | 0.711 |
| norank_o__Solirubrobacterales | 0.00±0.01 | 0.01±0.01 | 0.00±0.01 | 0.057 | 0.279 |
| A4b | 0.00±0.01 | 0.01±0.02 | 0.00±0.00 | 0.223 | 0.537 |
| norank_o__GCA004 | 0.01±0.05 | 0.00±0.00 | 0.00±0.00 | 0.328 | 0.568 |
| norank_o__C20 | 0.01±0.04 | 0.00±0.00 | 0.00±0.00 | 0.288 | 0.568 |
| [Acidaminobacteraceae] | 0.00±0.00 | 0.01±0.02 | 0.00±0.01 | 0.043 | 0.240 |
| norank_o__PK329 | 0.01±0.04 | 0.00±0.00 | 0.00±0.01 | 0.663 | 0.749 |
| norank_c__BD4-9 | 0.01±0.03 | 0.00±0.00 | 0.00±0.01 | 0.599 | 0.703 |
| norank_c__Gemmatimonadetes | 0.00±0.01 | 0.00±0.00 | 0.01±0.02 | 0.548 | 0.661 |
| Syntrophorhabdaceae | 0.01±0.04 | 0.00±0.01 | 0.00±0.00 | 0.611 | 0.709 |
| [Chromatiaceae] | 0.00±0.01 | 0.00±0.01 | 0.00±0.01 | 0.825 | 0.868 |
| norank_o__Lactobacillales | 0.01±0.03 | 0.00±0.00 | 0.00±0.01 | 0.431 | 0.624 |
| norank_o__EW055 | 0.00±0.01 | 0.00±0.01 | 0.00±0.01 | 0.047 | 0.249 |
| Nitrospiraceae | 0.00±0.01 | 0.01±0.01 | 0.00±0.00 | 0.001 | 0.016 |
| norank_c__SBRH58 | 0.00±0.01 | 0.01±0.01 | 0.00±0.01 | 0.716 | 0.803 |
| norank_c__ABS-6 | 0.00±0.01 | 0.01±0.02 | 0.00±0.00 | 0.324 | 0.568 |
| norank_o__pLW-97 | 0.00±0.01 | 0.00±0.01 | 0.00±0.00 | 0.010 | 0.088 |
| norank_o__S-BQ2-57 | 0.00±0.02 | 0.00±0.01 | 0.00±0.01 | 0.522 | 0.636 |
| norank_o__H39 | 0.00±0.02 | 0.01±0.01 | 0.00±0.00 | 0.019 | 0.133 |
| Caldilineaceae | 0.00±0.01 | 0.00±0.01 | 0.00±0.01 | 0.347 | 0.586 |
| Sporolactobacillaceae | 0.00±0.00 | 0.01±0.03 | 0.00±0.00 | 0.310 | 0.568 |
| [Entotheonellaceae] | 0.00±0.00 | 0.01±0.03 | 0.00±0.00 | 0.079 | 0.281 |
| norank_o__Euglenozoa | 0.00±0.00 | 0.01±0.01 | 0.00±0.00 | 0.008 | 0.070 |
| norank_o__Methylophilales | 0.00±0.00 | 0.01±0.03 | 0.00±0.00 | 0.324 | 0.568 |
| Gallionellaceae | 0.00±0.01 | 0.00±0.01 | 0.00±0.01 | 0.250 | 0.553 |
| Parachlamydiaceae | 0.00±0.02 | 0.00±0.01 | 0.00±0.00 | 0.280 | 0.568 |
| norank_o__PK29 | 0.00±0.00 | 0.01±0.02 | 0.00±0.00 | 0.168 | 0.469 |
| norank_o__Legionellales | 0.00±0.00 | 0.01±0.02 | 0.00±0.00 | 0.111 | 0.350 |
| norank_o__KD8-87 | 0.00±0.02 | 0.00±0.01 | 0.00±0.00 | 0.513 | 0.627 |
| **Appendix S2 (Continued)** |  |  |  |  |  |
|  | **Relative abundance （%）** | | | **N = 73** | |
| **Species name** | **adult** | **hindlimb bud stage** | **forelimb bud stage** | **Pvalue** | **Corrected pvalue** |
| Listeriaceae | 0.00±0.00 | 0.00±0.00 | 0.01±0.03 | 0.314 | 0.568 |
| norank_o__32-20 | 0.01±0.02 | 0.00±0.00 | 0.00±0.01 | 0.878 | 0.910 |
| norank_c__Thermoleophilia | 0.00±0.02 | 0.00±0.00 | 0.00±0.01 | 0.483 | 0.624 |
| norank_p__GAL15 | 0.01±0.03 | 0.00±0.00 | 0.00±0.00 | 0.110 | 0.350 |
| norank_o__SHA-20 | 0.00±0.00 | 0.00±0.00 | 0.01±0.03 | 0.575 | 0.685 |
| unclassified_o__Acidimicrobiales | 0.00±0.01 | 0.00±0.01 | 0.00±0.01 | 0.231 | 0.537 |
| norank_o__mle1-48 | 0.00±0.01 | 0.01±0.01 | 0.00±0.00 | 0.053 | 0.268 |
| [Fimbriimonadaceae] | 0.00±0.01 | 0.01±0.01 | 0.00±0.00 | 0.312 | 0.568 |
| mb2424 | 0.00±0.00 | 0.01±0.02 | 0.00±0.00 | 0.089 | 0.308 |
| norank_p__Chlorobi | 0.00±0.02 | 0.00±0.01 | 0.00±0.00 | 0.313 | 0.568 |
| unclassified_o__Cytophagales | 0.00±0.00 | 0.00±0.01 | 0.00±0.01 | 0.107 | 0.348 |
| Iamiaceae | 0.00±0.00 | 0.01±0.02 | 0.00±0.00 | 0.054 | 0.271 |
| norank_c__RB25 | 0.00±0.02 | 0.00±0.00 | 0.00±0.01 | 0.983 | 0.985 |
| Hyphomonadaceae | 0.00±0.01 | 0.00±0.00 | 0.00±0.01 | 0.117 | 0.369 |
| norank_c__SJA-28 | 0.01±0.03 | 0.00±0.00 | 0.00±0.00 | 0.601 | 0.704 |
| unclassified_o__Methylococcales | 0.00±0.01 | 0.00±0.01 | 0.00±0.01 | 0.531 | 0.642 |
| norank_c__3BR-5F | 0.01±0.04 | 0.00±0.00 | 0.00±0.00 | 0.508 | 0.624 |
| Cyclobacteriaceae | 0.00±0.00 | 0.01±0.02 | 0.00±0.00 | 0.006 | 0.055 |
| norank_o__[Saprospirales] | 0.00±0.00 | 0.00±0.01 | 0.00±0.01 | 0.291 | 0.568 |
| Anaeroplasmataceae | 0.00±0.00 | 0.00±0.01 | 0.00±0.02 | 0.314 | 0.568 |
| norank_o__Sediment-1 | 0.00±0.01 | 0.00±0.01 | 0.00±0.01 | 0.751 | 0.829 |
| Trueperaceae | 0.00±0.01 | 0.00±0.01 | 0.00±0.01 | 0.911 | 0.935 |
| Thermoactinomycetaceae | 0.00±0.01 | 0.01±0.02 | 0.00±0.00 | 0.048 | 0.249 |
| Bacteroidaceae_o__Bacteroidales_c__Bacteroidia | 0.00±0.00 | 0.01±0.02 | 0.00±0.00 | 0.316 | 0.568 |
| unclassified_o__SBR1031 | 0.00±0.00 | 0.00±0.01 | 0.00±0.01 | 0.187 | 0.499 |
| Gordoniaceae | 0.00±0.00 | 0.00±0.01 | 0.00±0.00 | 0.088 | 0.307 |
| norank_p__FCPU426 | 0.00±0.01 | 0.00±0.01 | 0.00±0.00 | 0.307 | 0.568 |
| norank_c__S085 | 0.00±0.01 | 0.00±0.00 | 0.00±0.02 | 0.923 | 0.943 |
| norank_c__SM2F11 | 0.00±0.01 | 0.00±0.01 | 0.00±0.00 | 0.194 | 0.515 |
| norank_o__AKYG1722 | 0.00±0.02 | 0.00±0.00 | 0.00±0.00 | 0.201 | 0.525 |
| mitochondria | 0.00±0.00 | 0.00±0.00 | 0.00±0.02 | 0.636 | 0.723 |
| Haliangiaceae | 0.00±0.01 | 0.00±0.01 | 0.00±0.00 | 0.075 | 0.281 |
| RB40 | 0.00±0.01 | 0.00±0.01 | 0.00±0.01 | 0.201 | 0.525 |
| norank_o__B12-WMSP1 | 0.00±0.00 | 0.00±0.01 | 0.00±0.00 | 0.001 | 0.016 |
| unclassified_p__Cyanobacteria | 0.00±0.00 | 0.01±0.01 | 0.00±0.00 | 0.000 | 0.008 |
| SHA-31 | 0.00±0.01 | 0.00±0.00 | 0.00±0.02 | 0.389 | 0.624 |
| norank_o__PHOS-HD29 | 0.00±0.00 | 0.00±0.00 | 0.01±0.02 | 0.575 | 0.685 |
| SB-1 | 0.00±0.00 | 0.00±0.00 | 0.00±0.03 | 0.818 | 0.864 |
| norank_c__B142 | 0.00±0.00 | 0.00±0.00 | 0.00±0.02 | 0.755 | 0.830 |
| unclassified_c__Chlamydiia | 0.00±0.00 | 0.00±0.01 | 0.00±0.00 | 0.029 | 0.172 |
| unclassified_c__Ktedonobacteria | 0.00±0.00 | 0.00±0.01 | 0.00±0.00 | 0.016 | 0.121 |
| Synergistaceae | 0.00±0.00 | 0.00±0.01 | 0.00±0.01 | 0.537 | 0.649 |
| Geodermatophilaceae | 0.00±0.00 | 0.00±0.00 | 0.00±0.02 | 0.660 | 0.748 |
| Pasteurellaceae | 0.00±0.00 | 0.00±0.00 | 0.00±0.01 | 0.817 | 0.864 |
| **Appendix S2 (Continued)** |  |  |  |  |  |
|  | **Relative abundance （%）** | | | **N = 73** | |
| **Species name** | **adult** | **hindlimb bud stage** | **forelimb bud stage** | **Pvalue** | **Corrected pvalue** |
| Caldicellulosiruptoraceae | 0.00±0.00 | 0.01±0.02 | 0.00±0.00 | 0.079 | 0.281 |
| norank_o__WCHB1-41 | 0.00±0.01 | 0.00±0.01 | 0.00±0.01 | 0.911 | 0.935 |
| Xenococcaceae | 0.00±0.01 | 0.00±0.00 | 0.00±0.01 | 0.317 | 0.568 |
| norank_o__Rhodospirillales | 0.00±0.00 | 0.00±0.00 | 0.00±0.02 | 0.319 | 0.568 |
| BS11 | 0.00±0.00 | 0.00±0.00 | 0.00±0.02 | 0.234 | 0.537 |
| norank_c__BB34 | 0.00±0.02 | 0.00±0.00 | 0.00±0.00 | 0.124 | 0.373 |
| [Barnesiellaceae] | 0.00±0.02 | 0.00±0.00 | 0.00±0.00 | 0.028 | 0.172 |
| [Cerasicoccaceae] | 0.00±0.00 | 0.00±0.01 | 0.00±0.00 | 0.107 | 0.348 |
| norank_o__SM1D11 | 0.00±0.00 | 0.00±0.01 | 0.00±0.00 | 0.316 | 0.568 |
| Shewanellaceae | 0.00±0.01 | 0.00±0.00 | 0.00±0.01 | 0.175 | 0.475 |
| SHA-116 | 0.00±0.01 | 0.00±0.00 | 0.00±0.01 | 0.818 | 0.864 |
| norank_o__AKAU3564 | 0.00±0.01 | 0.00±0.00 | 0.00±0.01 | 0.613 | 0.709 |
| norank_o__MND1 | 0.00±0.01 | 0.00±0.01 | 0.00±0.01 | 0.328 | 0.568 |
| Helicobacteraceae | 0.00±0.01 | 0.00±0.00 | 0.00±0.00 | 0.070 | 0.281 |
| Gemellaceae | 0.00±0.00 | 0.00±0.01 | 0.00±0.01 | 0.842 | 0.880 |
| norank_o__IIb | 0.00±0.02 | 0.00±0.00 | 0.00±0.00 | 0.328 | 0.568 |
| norank_o__Cryptophyta | 0.00±0.00 | 0.00±0.01 | 0.00±0.00 | 0.467 | 0.624 |
| norank_o__Dehalococcoidales | 0.00±0.00 | 0.00±0.01 | 0.00±0.00 | 0.316 | 0.568 |
| norank_o__FAC87 | 0.00±0.00 | 0.00±0.01 | 0.00±0.01 | 0.326 | 0.568 |
| Thermotogaceae | 0.00±0.00 | 0.00±0.01 | 0.00±0.00 | 0.088 | 0.307 |
| norank_c__PAUC37f | 0.00±0.02 | 0.00±0.00 | 0.00±0.00 | 0.508 | 0.624 |
| norank_c__OP8_1 | 0.00±0.02 | 0.00±0.00 | 0.00±0.00 | 0.333 | 0.568 |
| Rhodothermaceae | 0.00±0.00 | 0.00±0.01 | 0.00±0.00 | 0.079 | 0.281 |
| norank_o__RF39 | 0.00±0.00 | 0.00±0.00 | 0.00±0.00 | 0.121 | 0.370 |
| norank_o__NB1-j | 0.00±0.01 | 0.00±0.00 | 0.00±0.00 | 0.352 | 0.593 |
| norank_c__Gemm-3 | 0.00±0.01 | 0.00±0.01 | 0.00±0.00 | 0.102 | 0.337 |
| norank_o__Chlamydiales | 0.00±0.01 | 0.00±0.01 | 0.00±0.00 | 0.328 | 0.568 |
| unclassified_c__Chloroplast | 0.00±0.00 | 0.00±0.00 | 0.00±0.01 | 0.063 | 0.281 |
| unclassified_c__Acidobacteria-6 | 0.00±0.00 | 0.00±0.00 | 0.00±0.02 | 0.488 | 0.624 |
| unclassified_p__Acidobacteria | 0.00±0.00 | 0.00±0.00 | 0.00±0.01 | 0.818 | 0.864 |
| Caldicoprobacteraceae | 0.00±0.00 | 0.00±0.01 | 0.00±0.00 | 0.212 | 0.537 |
| Armatimonadaceae | 0.00±0.00 | 0.00±0.01 | 0.00±0.00 | 0.079 | 0.281 |
| ACK-M1 | 0.00±0.01 | 0.00±0.00 | 0.00±0.00 | 0.988 | 0.988 |
| norank_o__Gemmatimonadales | 0.00±0.00 | 0.00±0.01 | 0.00±0.01 | 0.326 | 0.568 |
| Alteromonadaceae | 0.00±0.01 | 0.00±0.00 | 0.00±0.01 | 0.783 | 0.857 |
| norank_c__Chloroplast | 0.00±0.00 | 0.00±0.00 | 0.00±0.01 | 0.234 | 0.537 |
| unclassified_c__Gemmatimonadetes | 0.00±0.00 | 0.00±0.00 | 0.00±0.01 | 0.818 | 0.864 |
| norank_o__FAC88 | 0.00±0.01 | 0.00±0.00 | 0.00±0.00 | 0.132 | 0.387 |
| norank_o__AKIW781 | 0.00±0.00 | 0.00±0.01 | 0.00±0.00 | 0.006 | 0.055 |
| norank_o__HA64 | 0.00±0.00 | 0.00±0.01 | 0.00±0.00 | 0.078 | 0.281 |
| Tsukamurellaceae | 0.00±0.00 | 0.00±0.01 | 0.00±0.00 | 0.328 | 0.568 |
| norank_o__A89 | 0.00±0.01 | 0.00±0.00 | 0.00±0.00 | 0.333 | 0.568 |
| Pseudanabaenaceae | 0.00±0.01 | 0.00±0.00 | 0.00±0.01 | 0.751 | 0.829 |
| norank_o__[Brevinematales] | 0.00±0.01 | 0.00±0.00 | 0.00±0.00 | 0.333 | 0.568 |
| **Appendix S2 (Continued)** |  |  |  |  |  |
|  | **Relative abundance （%）** | | | **N = 73** | |
| **Species name** | **adult** | **hindlimb bud stage** | **forelimb bud stage** | **Pvalue** | **Corrected pvalue** |
| norank_o__CFB-26 | 0.00±0.00 | 0.00±0.00 | 0.00±0.01 | 0.818 | 0.864 |
| unclassified_o__TG3-1 | 0.00±0.00 | 0.00±0.00 | 0.00±0.01 | 0.818 | 0.864 |
| norank_c__OP8_2 | 0.00±0.00 | 0.00±0.01 | 0.00±0.00 | 0.316 | 0.568 |
| unclassified_o__Solibacterales | 0.00±0.01 | 0.00±0.00 | 0.00±0.00 | 0.253 | 0.553 |
| Sphaerochaetaceae | 0.00±0.00 | 0.00±0.00 | 0.00±0.01 | 0.234 | 0.537 |
| Cystobacterineae | 0.00±0.00 | 0.00±0.01 | 0.00±0.00 | 0.006 | 0.055 |
| [Pedosphaeraceae] | 0.00±0.00 | 0.00±0.01 | 0.00±0.00 | 0.006 | 0.055 |
| norank_o__Phycisphaerales | 0.00±0.00 | 0.00±0.00 | 0.00±0.01 | 0.725 | 0.811 |
| Brocadiaceae | 0.00±0.01 | 0.00±0.00 | 0.00±0.00 | 0.231 | 0.537 |
| norank_o__Cytophagales | 0.00±0.00 | 0.00±0.00 | 0.00±0.01 | 0.326 | 0.568 |
| Cardiobacteriaceae | 0.00±0.00 | 0.00±0.00 | 0.00±0.01 | 0.488 | 0.624 |
| norank_o__S1198 | 0.00±0.00 | 0.00±0.00 | 0.00±0.01 | 0.626 | 0.715 |
| Desulfarculaceae | 0.00±0.00 | 0.00±0.00 | 0.00±0.01 | 0.488 | 0.624 |
| norank_o__SB-34 | 0.00±0.00 | 0.00±0.00 | 0.00±0.01 | 0.234 | 0.537 |
| norank_c__TK17 | 0.00±0.00 | 0.00±0.00 | 0.00±0.01 | 0.626 | 0.715 |
| unclassified_p__Chloroflexi | 0.00±0.00 | 0.00±0.01 | 0.00±0.00 | 0.048 | 0.249 |
| norank_o__GIF9 | 0.00±0.01 | 0.00±0.00 | 0.00±0.00 | 0.508 | 0.624 |
| Dethiosulfovibrionaceae | 0.00±0.00 | 0.00±0.00 | 0.00±0.00 | 0.754 | 0.830 |
| norank_o__LD1-PB3 | 0.00±0.01 | 0.00±0.00 | 0.00±0.00 | 0.332 | 0.568 |
| Williamsiaceae | 0.00±0.00 | 0.00±0.00 | 0.00±0.00 | 0.019 | 0.136 |
| norank_o__Thermomicrobiales | 0.00±0.00 | 0.00±0.00 | 0.00±0.01 | 0.488 | 0.624 |
| norank_o__Ellin5290 | 0.00±0.00 | 0.00±0.01 | 0.00±0.00 | 0.048 | 0.249 |
| norank_o__Alteromonadales | 0.00±0.00 | 0.00±0.00 | 0.00±0.01 | 0.335 | 0.570 |
| Leptospiraceae | 0.00±0.00 | 0.00±0.00 | 0.00±0.00 | 0.175 | 0.475 |
| norank_o__BPC076 | 0.00±0.01 | 0.00±0.00 | 0.00±0.00 | 0.613 | 0.709 |
| EB1003 | 0.00±0.01 | 0.00±0.00 | 0.00±0.00 | 0.333 | 0.568 |
| norank_o__SBla14 | 0.00±0.00 | 0.00±0.00 | 0.00±0.00 | 0.092 | 0.314 |
| norank_p__WS4 | 0.00±0.00 | 0.00±0.00 | 0.00±0.00 | 0.163 | 0.456 |
| norank_p__Fibrobacteres | 0.00±0.00 | 0.00±0.01 | 0.00±0.00 | 0.079 | 0.281 |
| unclassified_o__Acholeplasmatales | 0.00±0.00 | 0.00±0.00 | 0.00±0.00 | 0.209 | 0.535 |
| Actinospicaceae | 0.00±0.00 | 0.00±0.00 | 0.00±0.00 | 0.053 | 0.268 |
| 0319-6A21 | 0.00±0.01 | 0.00±0.00 | 0.00±0.00 | 0.370 | 0.617 |
| unclassified_p__LCP-89 | 0.00±0.01 | 0.00±0.00 | 0.00±0.00 | 0.253 | 0.553 |
| norank_o__SJA-22 | 0.00±0.01 | 0.00±0.00 | 0.00±0.00 | 0.613 | 0.709 |
| Dolo_23 | 0.00±0.00 | 0.00±0.00 | 0.00±0.01 | 0.326 | 0.568 |
| unclassified_o__Rickettsiales | 0.00±0.00 | 0.00±0.00 | 0.00±0.00 | 0.901 | 0.929 |
| Nitrosomonadaceae | 0.00±0.00 | 0.00±0.00 | 0.00±0.00 | 0.208 | 0.535 |
| norank_o__TG3-1 | 0.00±0.00 | 0.00±0.00 | 0.00±0.00 | 0.096 | 0.325 |
| unclassified_o__Phycisphaerales | 0.00±0.01 | 0.00±0.00 | 0.00±0.00 | 0.508 | 0.624 |
| norank_c__VC2_1_Bac22 | 0.00±0.00 | 0.00±0.01 | 0.00±0.00 | 0.079 | 0.281 |
| unclassified_o_Methylacidiphilales | 0.00±0.00 | 0.00±0.01 | 0.00±0.00 | 0.079 | 0.281 |
| norank_c__JG37-AG-4 | 0.00±0.00 | 0.00±0.00 | 0.00±0.00 | 0.333 | 0.568 |
| RFP12 | 0.00±0.00 | 0.00±0.00 | 0.00±0.01 | 0.234 | 0.537 |
| norank_o__PeHg47 | 0.00±0.00 | 0.00±0.00 | 0.00±0.00 | 0.316 | 0.568 |
| norank_c__Mb-NB09 | 0.00±0.00 | 0.00±0.00 | 0.00±0.00 | 0.316 | 0.568 |
| **Appendix S2 (Continued)** |  |  |  |  |  |
|  | **Relative abundance （%）** | | | **N = 73** | |
| **Species name** | **adult** | **hindlimb bud stage** | **forelimb bud stage** | **Pvalue** | **Corrected pvalue** |
| norank_o__Nitrosomonadales | 0.00±0.00 | 0.00±0.00 | 0.00±0.00 | 0.048 | 0.249 |
| norank_p__Acidobacteria | 0.00±0.00 | 0.00±0.00 | 0.00±0.01 | 0.326 | 0.568 |
| unclassified_o__Dehalococcoidales | 0.00±0.00 | 0.00±0.00 | 0.00±0.00 | 0.333 | 0.568 |
| Nocardiopsaceae | 0.00±0.00 | 0.00±0.00 | 0.00±0.00 | 0.228 | 0.537 |
| norank_o__MVP-88 | 0.00±0.00 | 0.00±0.00 | 0.00±0.00 | 0.310 | 0.568 |
| norank_o__DS-18 | 0.00±0.00 | 0.00±0.00 | 0.00±0.00 | 0.310 | 0.568 |
| norank_p__SC4 | 0.00±0.00 | 0.00±0.00 | 0.00±0.00 | 0.316 | 0.568 |
| norank_o__Haptophyceae | 0.00±0.00 | 0.00±0.00 | 0.00±0.00 | 0.589 | 0.694 |
| 211ds20 | 0.00±0.00 | 0.00±0.00 | 0.00±0.00 | 0.207 | 0.535 |
| Rubrobacteraceae | 0.00±0.00 | 0.00±0.00 | 0.00±0.00 | 0.059 | 0.281 |
| norank_o__NKB15 | 0.00±0.01 | 0.00±0.00 | 0.00±0.00 | 0.508 | 0.624 |
| norank_c__Chlamydiia | 0.00±0.00 | 0.00±0.00 | 0.00±0.00 | 0.324 | 0.568 |
| norank_o__Bacillales | 0.00±0.00 | 0.00±0.00 | 0.00±0.01 | 0.488 | 0.624 |
| Rivulariaceae | 0.00±0.00 | 0.00±0.00 | 0.00±0.01 | 0.488 | 0.624 |
| Hydrogenophilaceae | 0.00±0.00 | 0.00±0.00 | 0.00±0.00 | 0.582 | 0.691 |
| norank_c__KIST-JJY010 | 0.00±0.00 | 0.00±0.00 | 0.00±0.00 | 0.819 | 0.864 |
| norank_c__Acidobacteria-5 | 0.00±0.01 | 0.00±0.00 | 0.00±0.00 | 0.253 | 0.553 |
| unclassified_o__Chromatiales | 0.00±0.01 | 0.00±0.00 | 0.00±0.00 | 0.508 | 0.624 |
| norank_c__Anaerolineae | 0.00±0.01 | 0.00±0.00 | 0.00±0.00 | 0.508 | 0.624 |
| norank_c__TM1 | 0.00±0.01 | 0.00±0.00 | 0.00±0.00 | 0.508 | 0.624 |
| Solirubrobacteraceae | 0.00±0.01 | 0.00±0.00 | 0.00±0.00 | 0.508 | 0.624 |
| norank_p__GN02 | 0.00±0.01 | 0.00±0.00 | 0.00±0.00 | 0.508 | 0.624 |
| norank_o__PL-11B10 | 0.00±0.00 | 0.00±0.00 | 0.00±0.00 | 0.316 | 0.568 |
| AKIW659 | 0.00±0.00 | 0.00±0.00 | 0.00±0.00 | 0.330 | 0.568 |
| norank_c__PRR-11 | 0.00±0.00 | 0.00±0.00 | 0.00±0.00 | 0.332 | 0.568 |
| Nitriliruptoraceae | 0.00±0.00 | 0.00±0.00 | 0.00±0.01 | 0.488 | 0.624 |
| unclassified_o__Fusobacteriales | 0.00±0.00 | 0.00±0.00 | 0.00±0.00 | 0.211 | 0.537 |
| RF16 | 0.00±0.00 | 0.00±0.00 | 0.00±0.01 | 0.488 | 0.624 |
| norank_o__HOC36 | 0.00±0.00 | 0.00±0.00 | 0.00±0.00 | 0.207 | 0.535 |
| norank_c__Gammaproteobacteria | 0.00±0.00 | 0.00±0.00 | 0.00±0.00 | 0.333 | 0.568 |
| norank_c__At12OctB3 | 0.00±0.01 | 0.00±0.00 | 0.00±0.00 | 0.819 | 0.864 |
| norank_c__SHA-109 | 0.00±0.01 | 0.00±0.00 | 0.00±0.00 | 0.508 | 0.624 |
| Gemmatimonadaceae | 0.00±0.01 | 0.00±0.00 | 0.00±0.00 | 0.508 | 0.624 |
| AKIW874 | 0.00±0.01 | 0.00±0.00 | 0.00±0.00 | 0.508 | 0.624 |
| TSCOR003-O20 | 0.00±0.01 | 0.00±0.00 | 0.00±0.00 | 0.508 | 0.624 |
| norank_o__Blgi18 | 0.00±0.01 | 0.00±0.00 | 0.00±0.00 | 0.508 | 0.624 |
| S47 | 0.00±0.01 | 0.00±0.00 | 0.00±0.00 | 0.508 | 0.624 |
| unclassified_p__ZB3 | 0.00±0.00 | 0.00±0.00 | 0.00±0.00 | 0.333 | 0.568 |
| norank_o__I025 | 0.00±0.00 | 0.00±0.00 | 0.00±0.00 | 0.330 | 0.568 |
| norank_o__11-24 | 0.00±0.00 | 0.00±0.00 | 0.00±0.01 | 0.488 | 0.624 |
| norank_c__SHA-26 | 0.00±0.00 | 0.00±0.00 | 0.00±0.00 | 0.104 | 0.341 |
| [Melioribacteraceae] | 0.00±0.01 | 0.00±0.00 | 0.00±0.00 | 0.508 | 0.624 |
| norank_o__CL500-15 | 0.00±0.00 | 0.00±0.00 | 0.00±0.00 | 0.079 | 0.281 |
| Thiotrichaceae | 0.00±0.00 | 0.00±0.00 | 0.00±0.00 | 0.079 | 0.281 |
| norank_o__JH-WHS99 | 0.00±0.00 | 0.00±0.00 | 0.00±0.00 | 0.079 | 0.281 |
| **Appendix S2 (Continued)** |  |  |  |  |  |
|  | **Relative abundance （%）** | | | **N = 73** | |
| **Species name** | **adult** | **hindlimb bud stage** | **forelimb bud stage** | **Pvalue** | **Corrected pvalue** |
| norank_o__RsaHF231 | 0.00±0.00 | 0.00±0.00 | 0.00±0.00 | 0.079 | 0.281 |
| norank_o__MBA08 | 0.00±0.00 | 0.00±0.00 | 0.00±0.00 | 0.079 | 0.281 |
| norank_c__Ktedonobacteria | 0.00±0.00 | 0.00±0.00 | 0.00±0.00 | 0.330 | 0.568 |
| norank_o__wb1_H11 | 0.00±0.00 | 0.00±0.00 | 0.00±0.00 | 0.488 | 0.624 |
| Promicromonosporaceae | 0.00±0.00 | 0.00±0.00 | 0.00±0.00 | 0.234 | 0.537 |
| koll13 | 0.00±0.00 | 0.00±0.00 | 0.00±0.00 | 0.488 | 0.624 |
| norank_c__koll11 | 0.00±0.00 | 0.00±0.00 | 0.00±0.00 | 0.819 | 0.864 |
| norank_o__Fusobacteriales | 0.00±0.00 | 0.00±0.00 | 0.00±0.00 | 0.663 | 0.749 |
| unclassified_o__Chloroflexales | 0.00±0.00 | 0.00±0.00 | 0.00±0.00 | 0.102 | 0.337 |
| norank_p__Elusimicrobia | 0.00±0.00 | 0.00±0.00 | 0.00±0.00 | 0.508 | 0.624 |
| norank_o_Thermogemmatisporales | 0.00±0.00 | 0.00±0.00 | 0.00±0.00 | 0.253 | 0.553 |
| norank_c__TSBW08 | 0.00±0.00 | 0.00±0.00 | 0.00±0.00 | 0.508 | 0.624 |
| Streptosporangiaceae | 0.00±0.00 | 0.00±0.00 | 0.00±0.00 | 0.124 | 0.373 |
| norank_c__Mollicutes | 0.00±0.00 | 0.00±0.00 | 0.00±0.00 | 0.326 | 0.568 |
| norank_p__SBR1093 | 0.00±0.00 | 0.00±0.00 | 0.00±0.00 | 0.333 | 0.568 |
| norank_o__MVS-107 | 0.00±0.00 | 0.00±0.00 | 0.00±0.00 | 0.333 | 0.568 |
| unclassified_o__Bdellovibrionales | 0.00±0.00 | 0.00±0.00 | 0.00±0.00 | 0.488 | 0.624 |
| norank_p__CD12 | 0.00±0.00 | 0.00±0.00 | 0.00±0.00 | 0.488 | 0.624 |
| norank_p__OP8 | 0.00±0.00 | 0.00±0.00 | 0.00±0.00 | 0.819 | 0.864 |
| norank_c__BPC102 | 0.00±0.00 | 0.00±0.00 | 0.00±0.00 | 0.508 | 0.624 |
| norank_o__C114 | 0.00±0.00 | 0.00±0.00 | 0.00±0.00 | 0.253 | 0.553 |
| Gomphosphaeriaceae | 0.00±0.00 | 0.00±0.00 | 0.00±0.00 | 0.508 | 0.624 |
| norank_o__Synechococcales | 0.00±0.00 | 0.00±0.00 | 0.00±0.00 | 0.508 | 0.624 |
| norank_c__SHA-37 | 0.00±0.00 | 0.00±0.00 | 0.00±0.00 | 0.508 | 0.624 |
| norank_o__WCHB1-50 | 0.00±0.00 | 0.00±0.00 | 0.00±0.00 | 0.508 | 0.624 |
| norank_o__RB046 | 0.00±0.00 | 0.00±0.00 | 0.00±0.00 | 0.738 | 0.823 |
| unclassified_c__TK10 | 0.00±0.00 | 0.00±0.00 | 0.00±0.00 | 0.079 | 0.281 |
| unclassified_p__Actinobacteria | 0.00±0.00 | 0.00±0.00 | 0.00±0.00 | 0.079 | 0.281 |
| unclassified_o__[Rhodothermales] | 0.00±0.00 | 0.00±0.00 | 0.00±0.00 | 0.079 | 0.281 |
| Holosporaceae | 0.00±0.00 | 0.00±0.00 | 0.00±0.00 | 0.234 | 0.537 |
| unclassified_c__Elusimicrobia | 0.00±0.00 | 0.00±0.00 | 0.00±0.00 | 0.079 | 0.281 |
| norank_o__Methylacidiphilales | 0.00±0.00 | 0.00±0.00 | 0.00±0.00 | 0.079 | 0.281 |
| Caldithrixaceae | 0.00±0.00 | 0.00±0.00 | 0.00±0.00 | 0.079 | 0.281 |
| norank_c__SBZC_2415 | 0.00±0.00 | 0.00±0.00 | 0.00±0.00 | 0.079 | 0.281 |
| norank_p__BHI80-139 | 0.00±0.00 | 0.00±0.00 | 0.00±0.00 | 0.079 | 0.281 |
| AK1AB1_02E | 0.00±0.00 | 0.00±0.00 | 0.00±0.00 | 0.079 | 0.281 |
| norank_o__Ellin7246 | 0.00±0.00 | 0.00±0.00 | 0.00±0.00 | 0.079 | 0.281 |
| norank_o__DH61 | 0.00±0.00 | 0.00±0.00 | 0.00±0.00 | 0.488 | 0.624 |
| norank_o__agg27 | 0.00±0.00 | 0.00±0.00 | 0.00±0.00 | 0.079 | 0.281 |
| norank_o__BPC015 | 0.00±0.00 | 0.00±0.00 | 0.00±0.00 | 0.488 | 0.624 |
| norank_c__Endomicrobia | 0.00±0.00 | 0.00±0.00 | 0.00±0.00 | 0.079 | 0.281 |
| Succinivibrionaceae | 0.00±0.00 | 0.00±0.00 | 0.00±0.00 | 0.819 | 0.864 |
| unclassified_p__OD1 | 0.00±0.00 | 0.00±0.00 | 0.00±0.00 | 0.819 | 0.864 |
| norank_o__B07_WMSP1 | 0.00±0.00 | 0.00±0.00 | 0.00±0.00 | 0.620 | 0.711 |
| unclassified_p__TM7 | 0.00±0.00 | 0.00±0.00 | 0.00±0.00 | 0.819 | 0.864 |
| **Appendix S2 (Continued)** |  |  |  |  |  |
|  | **Relative abundance （%）** | | | **N = 73** | |
| **Species name** | **adult** | **hindlimb bud stage** | **forelimb bud stage** | **Pvalue** | **Corrected pvalue** |
| Criblamydiaceae | 0.00±0.00 | 0.00±0.00 | 0.00±0.00 | 0.253 | 0.553 |
| Synechococcaceae | 0.00±0.00 | 0.00±0.00 | 0.00±0.00 | 0.253 | 0.553 |
| norank_o__MSBL3 | 0.00±0.00 | 0.00±0.00 | 0.00±0.00 | 0.508 | 0.624 |
| norank_p__AC1 | 0.00±0.00 | 0.00±0.00 | 0.00±0.00 | 0.508 | 0.624 |
| norank_o__GW-28 | 0.00±0.00 | 0.00±0.00 | 0.00±0.00 | 0.508 | 0.624 |
| norank_c__HDBW-WB69 | 0.00±0.00 | 0.00±0.00 | 0.00±0.00 | 0.508 | 0.624 |
| norank_o__SBYZ_6080 | 0.00±0.00 | 0.00±0.00 | 0.00±0.00 | 0.508 | 0.624 |
| norank_c__GN15 | 0.00±0.00 | 0.00±0.00 | 0.00±0.00 | 0.508 | 0.624 |
| norank_o__Z20 | 0.00±0.00 | 0.00±0.00 | 0.00±0.00 | 0.508 | 0.624 |
| norank_c__SAW1_B44 | 0.00±0.00 | 0.00±0.00 | 0.00±0.00 | 0.253 | 0.553 |
| norank_o__DS-100 | 0.00±0.00 | 0.00±0.00 | 0.00±0.00 | 0.326 | 0.568 |
| norank_c__C0119 | 0.00±0.00 | 0.00±0.00 | 0.00±0.00 | 0.488 | 0.624 |
| unclassified_o__Lactobacillales | 0.00±0.00 | 0.00±0.00 | 0.00±0.00 | 0.110 | 0.350 |
| norank_o__Ucn15732 | 0.00±0.00 | 0.00±0.00 | 0.00±0.00 | 0.488 | 0.624 |
| unclassified_p__Planctomycetes | 0.00±0.00 | 0.00±0.00 | 0.00±0.00 | 0.508 | 0.624 |
| Sediment-4 | 0.00±0.00 | 0.00±0.00 | 0.00±0.00 | 0.508 | 0.624 |
| unclassified_p__OP3 | 0.00±0.00 | 0.00±0.00 | 0.00±0.00 | 0.508 | 0.624 |
| unclassified_c__TG3 | 0.00±0.00 | 0.00±0.00 | 0.00±0.00 | 0.508 | 0.624 |
| norank_o__GIF10 | 0.00±0.00 | 0.00±0.00 | 0.00±0.00 | 0.508 | 0.624 |
| norank_o__GMD14H09 | 0.00±0.00 | 0.00±0.00 | 0.00±0.00 | 0.508 | 0.624 |
| unclassified_c__Verruco-5 | 0.00±0.00 | 0.00±0.00 | 0.00±0.00 | 0.508 | 0.624 |
| Fibrobacteraceae | 0.00±0.00 | 0.00±0.00 | 0.00±0.00 | 0.318 | 0.568 |
| Thermoanaerobacteraceae | 0.00±0.00 | 0.00±0.00 | 0.00±0.00 | 0.488 | 0.624 |
| norank_o__M2PT2-76 | 0.00±0.00 | 0.00±0.00 | 0.00±0.00 | 0.488 | 0.624 |
| norank_c__iii1-8 | 0.00±0.00 | 0.00±0.00 | 0.00±0.00 | 0.488 | 0.624 |
| unclassified_o__[Roseiflexales] | 0.00±0.00 | 0.00±0.00 | 0.00±0.00 | 0.488 | 0.624 |
| unclassified_o__Ignavibacteriales | 0.00±0.00 | 0.00±0.00 | 0.00±0.00 | 0.508 | 0.624 |
| norank_c__BS119 | 0.00±0.00 | 0.00±0.00 | 0.00±0.00 | 0.508 | 0.624 |
| norank_p__NKB19 | 0.00±0.00 | 0.00±0.00 | 0.00±0.00 | 0.508 | 0.624 |
| norank_o__SSS58A | 0.00±0.00 | 0.00±0.00 | 0.00±0.00 | 0.508 | 0.624 |
| EtOH8 | 0.00±0.00 | 0.00±0.00 | 0.00±0.00 | 0.253 | 0.553 |
| norank_o__[Cloacamonales] | 0.00±0.00 | 0.00±0.00 | 0.00±0.00 | 0.508 | 0.624 |
| unclassified_c__Phycisphaerae | 0.00±0.00 | 0.00±0.00 | 0.00±0.00 | 0.508 | 0.624 |
| norank_o__CV90 | 0.00±0.00 | 0.00±0.00 | 0.00±0.00 | 0.508 | 0.624 |
| unclassified_p__KSB3 | 0.00±0.00 | 0.00±0.00 | 0.00±0.00 | 0.508 | 0.624 |
| norank_p__GOUTA4 | 0.00±0.00 | 0.00±0.00 | 0.00±0.00 | 0.508 | 0.624 |
| Chloroflexaceae | 0.00±0.00 | 0.00±0.00 | 0.00±0.00 | 0.508 | 0.624 |
| unclassified_c__Thermomicrobia | 0.00±0.00 | 0.00±0.00 | 0.00±0.00 | 0.508 | 0.624 |
| unclassified_p__Chlorobi | 0.00±0.00 | 0.00±0.00 | 0.00±0.00 | 0.508 | 0.624 |
| norank_o__VC38 | 0.00±0.00 | 0.00±0.00 | 0.00±0.00 | 0.508 | 0.624 |
| R4-41B | 0.00±0.00 | 0.00±0.00 | 0.00±0.00 | 0.508 | 0.624 |
| norank_o__[Roseiflexales] | 0.00±0.00 | 0.00±0.00 | 0.00±0.00 | 0.508 | 0.624 |
| norank_c__OS-K | 0.00±0.00 | 0.00±0.00 | 0.00±0.00 | 0.508 | 0.624 |
| norank_o__Chromatiales | 0.00±0.00 | 0.00±0.00 | 0.00±0.00 | 0.079 | 0.281 |
| Pasteuriaceae | 0.00±0.00 | 0.00±0.00 | 0.00±0.00 | 0.079 | 0.281 |
| **Appendix S2 (Continued)** |  |  |  |  |  |
|  | **Relative abundance （%）** | | | **N = 73** | |
| **Species name** | **adult** | **hindlimb bud stage** | **forelimb bud stage** | **Pvalue** | **Corrected pvalue** |
| unclassified_p__Elusimicrobia | 0.00±0.00 | 0.00±0.00 | 0.00±0.00 | 0.079 | 0.281 |
| Rhodobiaceae | 0.00±0.00 | 0.00±0.00 | 0.00±0.00 | 0.079 | 0.281 |
| norank_p__TM6 | 0.00±0.00 | 0.00±0.00 | 0.00±0.00 | 0.079 | 0.281 |
| unclassified_o__Flavobacteriales | 0.00±0.00 | 0.00±0.00 | 0.00±0.00 | 0.234 | 0.537 |
| unclassified_o__Chlorophyta | 0.00±0.00 | 0.00±0.00 | 0.00±0.00 | 0.488 | 0.624 |
| Rs-045 | 0.00±0.00 | 0.00±0.00 | 0.00±0.00 | 0.488 | 0.624 |
| Planctomycetaceae | 0.00±0.00 | 0.00±0.00 | 0.00±0.00 | 0.488 | 0.624 |
| norank_p__Cyanobacteria | 0.00±0.00 | 0.00±0.00 | 0.00±0.00 | 0.488 | 0.624 |
| norank_o__Herpetosiphonales | 0.00±0.00 | 0.00±0.00 | 0.00±0.00 | 0.508 | 0.624 |
| norank_c__Pla3 | 0.00±0.00 | 0.00±0.00 | 0.00±0.00 | 0.508 | 0.624 |
| At425_EubF1 | 0.00±0.00 | 0.00±0.00 | 0.00±0.00 | 0.508 | 0.624 |
| norank_c__Gitt-GS-136 | 0.00±0.00 | 0.00±0.00 | 0.00±0.00 | 0.508 | 0.624 |
| norank_c__0319-6E2 | 0.00±0.00 | 0.00±0.00 | 0.00±0.00 | 0.508 | 0.624 |
| norank_p__GN04 | 0.00±0.00 | 0.00±0.00 | 0.00±0.00 | 0.508 | 0.624 |
| norank_c__GKS2-174 | 0.00±0.00 | 0.00±0.00 | 0.00±0.00 | 0.508 | 0.624 |
| unclassified_c__Dehalococcoidetes | 0.00±0.00 | 0.00±0.00 | 0.00±0.00 | 0.508 | 0.624 |
| Bogoriellaceae | 0.00±0.00 | 0.00±0.00 | 0.00±0.00 | 0.508 | 0.624 |
| norank_c__OP11-3 | 0.00±0.00 | 0.00±0.00 | 0.00±0.00 | 0.488 | 0.624 |
| unclassified_p__AD3 | 0.00±0.00 | 0.00±0.00 | 0.00±0.00 | 0.488 | 0.624 |
| OM60 | 0.00±0.00 | 0.00±0.00 | 0.00±0.00 | 0.488 | 0.624 |
| Chlamydomonadaceae | 0.00±0.00 | 0.00±0.00 | 0.00±0.00 | 0.508 | 0.624 |
| norank_o__RB41 | 0.00±0.00 | 0.00±0.00 | 0.00±0.00 | 0.508 | 0.624 |
| [Kouleothrixaceae] | 0.00±0.00 | 0.00±0.00 | 0.00±0.00 | 0.508 | 0.624 |

**Appendix S3** Differences in KEGG Pathways Level 2

|  | **Relative abundance (%)** | | | **Mann-Whitney U test** | |
| --- | --- | --- | --- | --- | --- |
| **KEGG Pathway level2** | **forelimb bud stage** | **hindlimb bud stage** | **adult** | **z** | **p (FDR)** |
| Amino Acid Metabolism | 10.43±0.92 | 10.12±0.42 | 10.96±0.61 | -3.982 | 0.000 |
| Biosynthesis of Other Secondary Metabolites | 10.14±0.71 | 10.28±1.4 | 10.02±0.73 | -4.400 | 0.000 |
| Cancers | 5.15±0.42 | 5.36±0.54 | 5.65±0.43 | -2.150 | 0.032 |
| Carbohydrate Metabolism | 1.71±0.33 | 1.58±0.29 | 1.97±0.34 | -4.313 | 0.000 |
| Cardiovascular Diseases | 3.88±0.81 | 3.34±0.44 | 4.17±0.47 | -3.751 | 0.000 |
| Cell Communication | 3.16±0.47 | 3.58±0.34 | 3.01±0.33 | -0.056 | 0.955 |
| Cell Motility | 0.85±0.10 | 0.89±0.18 | 0.83±0.11 | -4.818 | 0.000 |
| Cellular Processes and Signaling | 0.11±0.01 | 0.11±0.04 | 0.18±0.08 | -4.703 | 0.000 |
| Circulatory System | 0.00±0.00 | 0.01±0.01 | 0.02±0.01 | -1.169 | 0.243 |
| Digestive System | 0.00±0.00 | 0.00±0.00 | 0.00±0.00 | -3.549 | 0.000 |
| Endocrine System | 4.02±0.42 | 3.89±0.22 | 3.64±0.37 | -3.866 | 0.000 |
| Energy Metabolism | 1.89±0.20 | 2.02±0.12 | 1.71±0.14 | -4.140 | 0.000 |
| Enzyme Families | 0.05±0.01 | 0.04±0.02 | 0.04±0.01 | -4.674 | 0.000 |
| Excretory System | 0.43±0.1 | 0.38±0.03 | 0.46±0.05 | -4.775 | 0.000 |
| Folding Sorting and Degradation | 0.08±0.01 | 0.08±0.01 | 0.08±0.01 | -4.212 | 0.000 |
| Genetic Information Processing | 2.73±0.15 | 2.57±0.09 | 2.44±0.32 | -4.256 | 0.000 |
| Glycan Biosynthesis and Metabolism | 3.91±0.26 | 4.03±0.18 | 3.9±0.19 | -4.775 | 0.000 |
| Immune System | 1.86±0.25 | 1.66±0.23 | 1.97±0.23 | -4.068 | 0.000 |
| Immune System Diseases | 2.07±0.46 | 1.84±0.23 | 2.28±0.24 | -4.256 | 0.000 |
| Infectious Diseases | 0.21±0.07 | 0.2±0.09 | 0.39±0.18 | -4.667 | 0.000 |
| Lipid Metabolism | 5.10±0.37 | 4.82±0.26 | 4.83±0.28 | -4.010 | 0.000 |
| **Appendix S3 (Continued)** |  |  |  |  |  |
|  | **Relative abundance (%)** | | | **Mann-Whitney U test** | |
| **KEGG Pathway level2** | **forelimb bud stage** | **hindlimb bud stage** | **adult** | **z** | **p (FDR)** |
| Membrane Transport | 0.41±0.06 | 0.47±0.04 | 0.53±0.12 | -3.953 | 0.000 |
| Metabolic Diseases | 2.98±0.64 | 3.20±0.85 | 2.65±0.49 | -4.515 | 0.000 |
| Metabolism | 0.25±0.07 | 0.25±0.08 | 0.3±0.05 | -4.429 | 0.000 |
| Metabolism of Cofactors and Vitamins | 0.03±0.02 | 0.02±0.02 | 0.07±0.04 | -4.645 | 0.000 |
| Metabolism of Terpenoids and Polyketides | 0.38±0.13 | 0.32±0.10 | 0.4±0.06 | -4.068 | 0.000 |
| Nervous System | 0.14±0.03 | 0.16±0.01 | 0.13±0.02 | -3.910 | 0.000 |
| Neurodegenerative Diseases | 0.04±0.01 | 0.04±0.01 | 0.03±0.01 | -4.010 | 0.000 |
| Nucleotide Metabolism | 0.06±0.01 | 0.07±0.01 | 0.06±0.02 | -0.361 | 0.718 |
| Poorly Characterized | 0.10±0.02 | 0.1±0.02 | 0.1±0.02 | -4.501 | 0.000 |
| Replication and Repair | 0.00±0.00 | 0.00±0.00 | 0.00±0.00 | -4.515 | 0.000 |
| Sensory System | 13.44±1.58 | 13.01±1.22 | 12.74±1.12 | -4.342 | 0.000 |
| Signal Transduction | 2.37±0.36 | 2.08±0.30 | 2.21±0.30 | -0.485 | 0.627 |
| Signaling Molecules and Interaction | 0.20±0.04 | 0.18±0.03 | 0.16±0.03 | -4.602 | 0.000 |
| Transcription | 2.08±0.14 | 2.21±0.09 | 2.2±0.21 | -4.833 | 0.000 |
| Translation | 6.75±1.17 | 8.01±0.84 | 6.85±1.04 | -4.833 | 0.000 |
| Transport and Catabolism | 2.64±0.31 | 2.99±0.40 | 2.30±0.30 | -4.270 | 0.000 |
| Xenobiotics Biodegradation and Metabolism | 4.09±0.81 | 4.83±0.50 | 4.28±0.78 | -3.462 | 0.001 |
